# Effects of 4 Interpretive Front-of-Package Labeling Systems on Hypothetical Beverage and Snack Selections: A Randomized Clinical Trial

**DOI:** 10.1001/jamanetworkopen.2023.33515

**Published:** 2023-09-13

**Authors:** Anna H. Grummon, Laura A. Gibson, Aviva A. Musicus, Alisa J. Stephens-Shields, Sophia V. Hua, Christina A. Roberto

**Affiliations:** 1 Department of Pediatrics, Stanford University School of Medicine, Palo Alto, California; 2 Department of Health Policy, Stanford University School of Medicine, Stanford, California; 3 Department of Medical Ethics and Healthy Policy, University of Pennsylvania Perelman School of Medicine, Philadelphia; 4 Department of Nutrition, Harvard T.H. Chan School of Public Health, Boston, Massachusetts; 5 Department of Biostatistics, Epidemiology and Informatics, University of Pennsylvania Perelman School of Medicine, Philadelphia; 6 Now with Center for Science in the Public Interest, Washington, DC

## Abstract

**Importance:**

Policymakers and researchers have proposed a variety of interpretative front-of-package food labeling systems, but it remains unclear which is most effective at encouraging people to choose healthier foods and beverages, including among people with less education.

**Objective:**

To test the effects of 4 interpretative front-of-package food labeling systems on the healthfulness of beverage and snack selections, overall and by education level.

**Design, Setting, and Participants:**

This randomized clinical trial of a national sample of US adults 18 years and older was conducted online from November 16 to December 3, 2022.

**Intervention:**

Participants were randomized to view products with 1 of 5 food labeling systems, including control (calorie labels only) or 1 of 4 interpretative labeling systems: green (“choose often”) labels added to healthy foods; single traffic light labels added to healthy, moderately healthy, and unhealthy foods; physical activity calorie equivalent labels added to all products; and nutrient warning labels added to products high in calories, sugar, saturated fat, or sodium. All conditions had calorie labels on all products.

**Main Outcomes and Measures:**

Participants selected 1 of 16 beverages and 1 of 16 snacks that they wanted to hypothetically purchase. The primary outcomes were calories selected from beverages and from snacks. Secondary outcomes included label reactions and perceptions.

**Results:**

A total of 7945 participants completed the experiment and were included in analyses (4078 [51%] female, 3779 [48%] male, and 88 [1%] nonbinary or another gender; mean [SD] age, 47.5 [17.9 years]). Compared with the control arm, exposure to the green (average differential effect [ADE], −34.2; 95% CI, −42.2 to −26.1), traffic light (ADE, −31.5; 95% CI, −39.5 to −23.4), physical activity (ADE, −39.0; 95% CI, −47.0 to −31.1), or nutrient warning labels (ADE, −28.2; 95% CI, −36.2 to −20.2) led participants to select fewer calories from beverages (all *P* < .001). Similarly, compared with the control label, exposure to the green (ADE, −12.7; 95% CI, −17.3 to −8.2), traffic light (ADE, −13.7; 95% CI, −18.2 to −9.1), physical activity (ADE, −18.5; 95% CI, −23.1 to −13.9), or nutrient warning labels (ADE, −14.2; 95% CI, −18.8 to −9.6) led participants to select fewer calories from snacks (all *P* < .001). These effects did not differ by education level. The green labels were rated as less stigmatizing than the other interpretative systems but otherwise generally received the least favorable label reactions and perceptions (eg, elicited less attention, were perceived as less trustworthy), while the nutrient warnings and physical activity labels received the most favorable ratings.

**Conclusions and Relevance:**

In this randomized clinical trial of front-of-package food labeling systems, all 4 interpretative labeling systems reduced calories selected from beverages and from snacks compared with calorie labels, with no differences by education level.

**Trial Registration:**

ClinicalTrials.gov Identifier: NCT05432271

## Key Points

**Question:**

What are the effects of 4 interpretative front-of-package food labeling systems on beverage and snack selections, and do effects differ by education level?

**Findings:**

In this randomized clinical trial of 7945 adults, participants exposed to green (“choose often”), single traffic light, physical activity calorie equivalent, or nutrient warning labels selected 28 to 39 fewer calories from beverages and 13 to 18 fewer calories from snacks than participants exposed to calorie labels, which were statistically significant reductions that were similar across the 4 interpretative labels. Effects did not differ by participants’ education level.

**Meaning:**

Interpretative food labeling systems can encourage selection of healthier products among people with varying education levels.

## Teaser

This randomized clinical trial tests the effects of 4 interpretative front-of-package food labeling systems on the healthfulness of beverage and snack selections overall and by education level.

## Introduction

Poor dietary quality remains a leading cause of death in the US.^1^ Although most US adults have suboptimal diets, dietary quality is even lower—and has improved more slowly—among people with lower education levels.^2^ One potential driver of poor dietary quality is that people have limited access to easy-to-understand nutrition information when deciding which foods to buy. Currently, the primary source of nutrition information in the US is the Nutrition Facts label.^3^ Although this label provides important information about nutritional content and ingredients, it typically appears on the back or side of product packaging and includes only numeric information (eg, calorie content). Perhaps for these reasons, many people—particularly people with lower education levels—have difficulty using and understanding this label.^4,5,6^

To provide easier-to-access nutrition information and address poor dietary quality, the Biden-Harris Administration and the US Food and Drug Administration (FDA) have indicated plans to develop a front-of-package food labeling system that would quickly communicate products’ nutritional quality.^7,8^ There is broad agreement that such food labeling systems should be *interpretative*—that is, they should provide guidance, judgment, or recommendations rather than only numeric information.^9,10,11^ Although evidence indicates that interpretative food labeling systems encourage people to make healthier food purchases,^10,11,12,13,14^ it is unclear which systems are most effective at changing behavior, including for people with lower education levels. This gap is important given that more than half of US adults have not completed a college degree.^15^

Several types of front-of-package food labeling systems have been proposed or studied. The FDA, for example, is developing an “endorsement” label that promotes foods meeting the FDA’s definition of healthy.^16,17^ Research has also suggested the promise of single traffic light labels that categorize foods and beverages as unhealthy (red labels), moderately healthy (yellow labels), or healthy (green labels)^18^ and physical activity calorie equivalent labels that translate foods’ calorie content into minutes of physical activity needed to burn those calories.^19^ Several Latin American countries require that products display nutrient warning labels on foods and beverages that are high in nutrients of concern, like sugar and sodium,^20^ while voluntary labeling systems, like Australia’s Health Star Rating and France’s Nutri-Score, rate products’ overall healthfulness.^21,22,23^ Because studies have not tested some of these labeling systems against one another, their relative effects on behavior are unknown, making it challenging for policymakers to know which labels to pursue. Moreover, it is unclear whether interpretative food labeling systems have different effects depending on people’s education level, as most randomized trials of food labeling systems have had limited power to detect moderation by education.

To address these gaps and inform food labeling policies, this randomized clinical trial sought to examine the effect of different food labeling systems on beverage and snack selections and to assess whether label effects differ by education level in a well-powered sample. We also evaluated how people responded to and perceived the labeling systems.

## Methods

### Participants

The survey research firm CloudResearch recruited a sample of US adults approximately matched to the US population on gender, age, ethnicity, and US Census Bureau region. Recruitment occurred from November 16 to December 3, 2022, with a pause during Thanksgiving weekend. Eligible participants lived in the US and were 18 years or older. To maximize statistical power to detect moderation by education level, approximately half of the sample had lower (some college or less) and higher (associate degree or higher) education levels.

The University of Pennsylvania Institutional Review Board approved the study, and participants provided electronic informed consent. We preregistered the study design and analysis plan prior to data collection (https://aspredicted.org/8e34a.pdf; Supplement 1). We followed the Consolidated Standards of Reporting Trials (CONSORT) guidelines for reporting clinical trials.

### Approach

**Figure 1.**
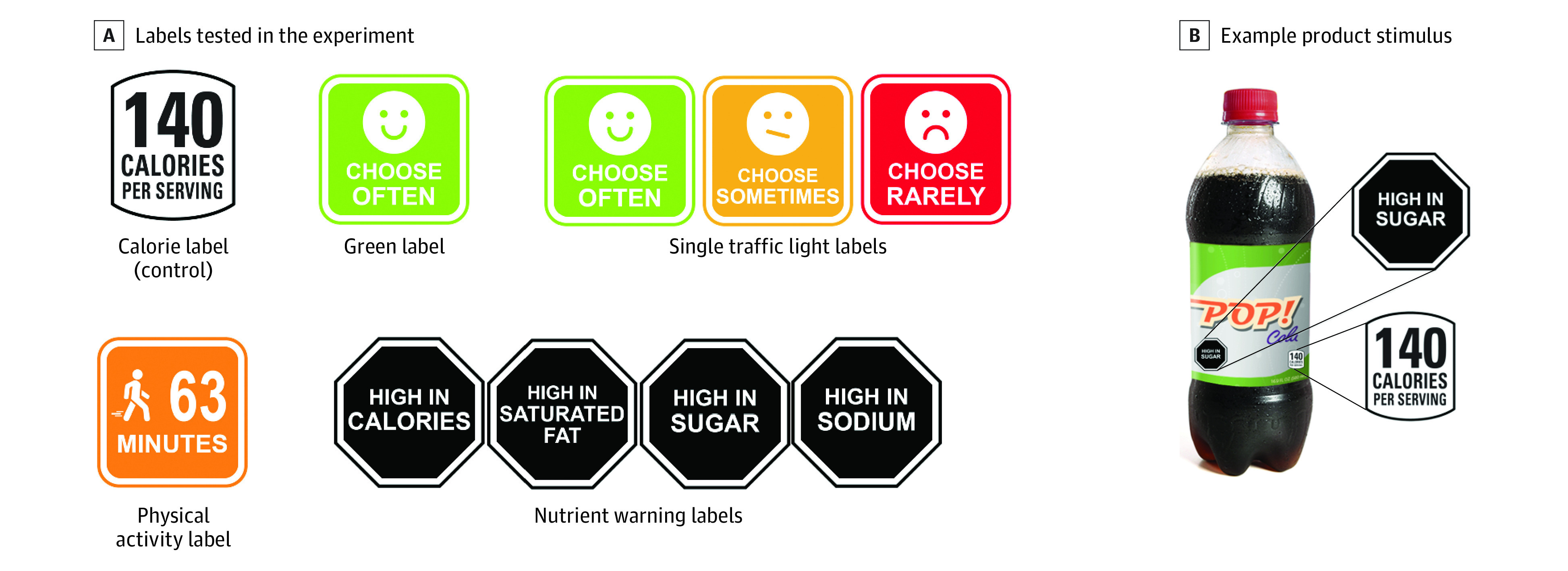
Labeling Systems Tested and Example Product Stimulus Used Participants were shown branded items with the labels shown in the figure.

Participants completed an online survey programmed in Qualtrics. Qualtrics randomized participants to 1 of 5 trial arms using a simple allocation ratio: (1) control (calorie labels only on all products); (2) green labels (green “choose often” labels added to healthy products with no additional labels on products not meeting healthfulness criteria); (3) single traffic light labels (hereafter, traffic light labels; green “choose often,” yellow “choose sometimes,” and red “choose rarely” labels added to healthy, moderately healthy, and unhealthy products, respectively); (4) physical activity labels (labels showing calorie content expressed as minutes of physical activity required to burn those calories added to all products); or (5) nutrient warning labels (“high in” warning labels added to products exceeding thresholds for sugar, sodium, saturated fat, or calories) (Figure 1A). Calorie labels were displayed in all arms because most beverages and packaged snacks in the US display these labels.^24^ Interpretative labels were added to the calorie labels in the interpretative labeling arms. We examined these 4 interpretative labeling systems to allow for comparison with the author team’s recent field experiment using the same systems^25^ and because they represent a range of types of interpretative labeling systems (one that only encourages healthy foods, one that only discourages unhealthy foods, etc). The eMethods in Supplement 2 detail criteria for assigning labels to each product. The calorie labels matched the design of the US Facts Up Front labels.^26^ The traffic light and physical activity labels were similar to those used in prior studies.^27,28,29^ The nutrient warnings were based on warnings mandated in several Latin American countries.^20^

### Procedures

After participants reviewed consent instructions and provided electronic informed consent via the survey they were instructed to complete 2 online vending machine selection tasks: one for beverages and one for snacks, in random order. We focused on vending machine selections to allow comparison with the author team’s field experiment,^25^ which also focused on vending machine selections (analyses ongoing at time of publication). Before viewing the products, participants in the interpretative labeling systems viewed a digital poster explaining their randomly assigned labeling system, modeled on posters used in the field experiment (eFigure in Supplement 2). Next, all participants were asked to imagine that they were viewing a vending machine and to select 1 item that they would most like to purchase from 16 products shown in random arrangement, similar to prior studies.^17,30,31^ eTable 1 in Supplement 2 summarizes product details. Products were shown with participants’ assigned label(s) on the front of the package. To ensure that the labels were visible, enlarged label(s) were displayed next to each product, similar to prior studies^17,30,31,32^ (Figure 1B).

To minimize social desirability bias and incentivize participants to select items that they wanted to receive, participants were told that 100 participants would be randomly chosen to have their selections delivered. In reality, these participants received a $2.50 gift card (more than the amount of the most expensive product); participants were debriefed on this procedure and could request that their data be removed. Participants were prompted (but not required) to select a product in each selection task. After completing the tasks, participants responded to survey questions.

### Measures

The primary outcomes were calories selected from beverages and from snacks. Secondary outcomes included likelihood of selecting a healthy, moderately healthy, or unhealthy item, and amount of sugar (g), sodium (mg), and saturated fat (g) selected. Secondary outcomes also included several psychological constructs associated with labels’ potential to change behavior,^13,33,34,35,36^ including label reactions (eg, attention, emotional reactions) and product perceptions (ie, perceived healthfulness). We also examined stigma^37,38^ and label perceptions^39,40^ (eg, trustworthiness). eTable 2 in Supplement 2 summarizes secondary psychological outcomes, and eTable 3 in Supplement 2 provides full survey measures.

### Statistical Analysis

The eMethods in Supplement 2 provide details on power calculations. Briefly, the target total sample size of 8000 participants (1600 per labeling arm) would yield 80% power to detect an effect of each interpretative labeling system vs control on beverage calories selected of Cohen *d* = 0.167 (approximately 11 kcal) or larger and 80% power to detect an interaction between the front-of-package labeling arm and education of Cohen *d* = 0.28 (approximately 18.5 kcal) or larger.

We preregistered all analyses except where noted (https://aspredicted.org/8e34a.pdf). All analyses were intent to treat. We examined the effect of each interpretative labeling arm on calories selected using regression. For beverage calories, we used 2-part models to address zero inflation, with logistic and ordinary least squares (OLS) regression for the first and secondary parts, respectively. For snack calories, we used OLS regression. All models regressed calories selected on indicator variables for each trial arm (excluding the control as the referent). No covariates were included. We used the models to estimate average differential effects (ADEs) of each interpretive labeling system compared with control. The ADEs for 2-part models were calculated overall (ie, combining the 2 parts of the model) using the chain rule; standard errors were calculated using the delta method. We used the Holm-Bonferroni method to correct *P* values for multiple comparisons, considering 4 tests per outcome (each interpretative labeling system vs control). We additionally tested whether the 4 interpretative labeling systems differed from one another in their effects on calories using χ^2^ tests (beverage calories) or Wald tests (snack calories). We corrected these *P *values using the Holm-Bonferroni method (6 tests to compare the 4 interpretive labeling systems to one another). Throughout, we report corrected *P* values.

We examined whether the effects of the interpretive labeling systems on calories selected were moderated by education by adding education level (some college or less vs any college degree) and its interaction with trial arms to the primary models. We examined the joint statistical significance of the interaction terms.

We followed a similar approach to examine secondary outcomes using 2-part models for zero-inflated continuous outcomes (ie, sugar), OLS for nonzero-inflated continuous outcomes (eg, sodium, attention to labels), logistic regression for binary outcomes (eg, noticing the label), and multinomial logistic regressions for categorical outcomes (eg, likelihood of selecting a healthy, moderately healthy, or unhealthy beverage; we opted against ordered logit due to lack of proportionality^41,42^). Comparisons of the interpretative labeling systems to one another for secondary outcomes were not preregistered.

We also conducted non-preregistered moderation analyses examining whether the effects of the interpretative labels on stigma were moderated by body mass index (calculated as weight in kilograms divided by height in meters squared; ≥30 kg/m^2^ vs <30 kg/m^2^) using the same approach as for the education moderation analyses. Analyses were conducted in Stata/MP, version 17.1 (StataCorp), and used 2-tailed tests. A corrected *P* < .05 was considered to be statistically significant. All analyses were replicated by a second analyst.

## Results

### Participant Characteristics

**Figure 2.**
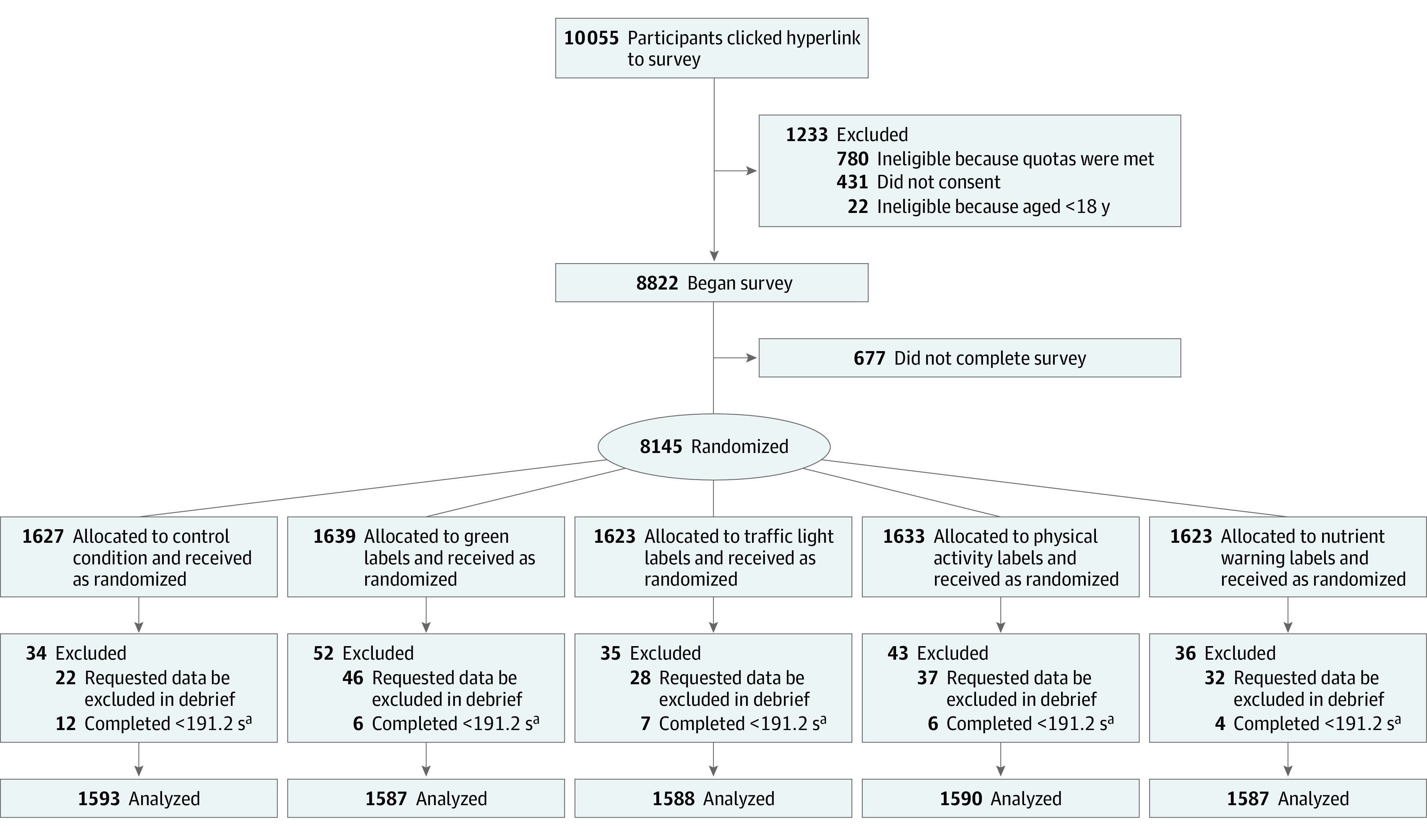
Participant Flow Diagram ^a^The time of 191.2 seconds indicates one-third of the median completion time in a soft launch of the survey. Likelihood of exclusion due to completing too quickly or requesting data be excluded in debrief did not differ across trial arms.

A total of 8822 participants were enrolled, and 7945 were included in the analytic sample (Figure 2; all exclusions were preregistered). The sample included 4078 (51%) women, 3779 (48%) men, and 88 (1%) individuals who were nonbinary or another gender, and the mean (SD) age among all was 47.5 (17.9) years (Table 1). The sample was similar to the US in distribution of gender, race and ethnicity, and self-reported dietary quality but included higher proportions of people with higher education and lower income (eTable 4 in Supplement 2).

**Table 1.** Participant Characteristics by Labeling System (N = 7945)^a^

Characteristic	No. (%)				
	Calorie labels (control) (n = 1593)	Green labels (n = 1587)	Single traffic light labels (n = 1588)	Physical activity labels (n = 1590)	Nutrient warning labels (n = 1587)
Age, y					
18-29	310 (19)	311 (20)	324 (20)	317 (20)	343 (22)
30-44	419 (26)	382 (24)	402 (25)	408 (26)	408 (26)
45-59	426 (27)	394 (25)	385 (24)	374 (24)	389 (25)
≥60	438 (27)	500 (32)	477 (30)	491 (31)	447 (28)
Gender					
Female	814 (51)	799 (50)	825 (52)	844 (53)	796 (50)
Male	757 (48)	773 (49)	746 (47)	729 (46)	774 (49)
Nonbinary or another gender	22 (1)	15 (1)	17 (1)	17 (1)	17 (1)
Race					
American Indian or Alaska Native	25 (2)	26 (2)	24 (2)	24 (2)	17 (1)
Asian or Pacific Islander	40 (3)	38 (2)	50 (3)	56 (4)	52 (3)
Black or African American	193 (12)	205 (13)	195 (12)	186 (12)	183 (12)
White	1208 (76)	1200 (76)	1211 (76)	1222 (77)	1221 (77)
Other or multiracial	126 (8)	118 (7)	108 (7)	102 (6)	114 (7)
Latino or Hispanic ethnicity	295 (19)	235 (15)	270 (17)	246 (15)	251 (16)
Education					
High school diploma or less	417 (26)	401 (25)	426 (27)	437 (27)	436 (27)
Some college	409 (26)	361 (23)	337 (21)	367 (23)	340 (21)
College graduate or associate degree	596 (37)	650 (41)	639 (40)	627 (39)	614 (39)
Graduate degree	171 (11)	175 (11)	186 (12)	159 (10)	197 (12)
Use of Nutrition Facts panel					
Never	194 (12)	166 (10)	195 (12)	169 (11)	180 (11)
Rarely	311 (20)	264 (17)	284 (18)	281 (18)	276 (17)
Sometimes	590 (37)	605 (38)	615 (39)	594 (37)	605 (38)
Most of the time	337 (21)	395 (25)	356 (22)	383 (24)	362 (23)
Always	160 (10)	157 (10)	138 (9)	163 (10)	164 (10)
Self-rated diet quality					
Poor	97 (6)	75 (5)	99 (6)	101 (6)	73 (5)
Fair	482 (30)	470 (30)	461 (29)	465 (29)	478 (30)
Good	640 (40)	628 (40)	630 (40)	672 (42)	646 (41)
Very good	290 (18)	322 (20)	286 (18)	270 (17)	295 (19)
Excellent	84 (5)	92 (6)	112 (7)	82 (5)	95 (6)
Household size					
1	342 (21)	386 (24)	351 (22)	340 (21)	342 (22)
2	531 (33)	545 (34)	574 (36)	578 (36)	544 (34)
3	315 (20)	295 (19)	275 (17)	294 (18)	297 (19)
≥4	405 (25)	361 (23)	387 (24)	378 (24)	404 (25)
No. of children					
0	1066 (67)	1128 (71)	1104 (70)	1105 (70)	1103 (70)
1	272 (17)	235 (15)	227 (14)	231 (15)	228 (14)
2	158 (10)	136 (9)	161 (10)	163 (10)	163 (10)
≥3	96 (6)	87 (5)	95 (6)	89 (6)	93 (6)
Annual household income, $					
0-24 999	370 (23)	427 (27)	413 (26)	425 (27)	407 (26)
25 000-49 999	498 (31)	453 (29)	458 (29)	440 (28)	467 (29)
50 000-74 999	309 (19)	283 (18)	311 (20)	304 (19)	299 (19)
≥75 000	416 (26)	422 (27)	406 (26)	415 (26)	411 (26)
Income ≤150% FPL	440 (28)	496 (31)	462 (29)	470 (30)	452 (29)
BMI					
Underweight (<18.5)	73 (5)	65 (4)	64 (4)	54 (3)	56 (4)
Healthy weight (18.5 to <25.0)	497 (31)	530 (33)	578 (36)	557 (35)	558 (35)
Overweight (25.0 to <30.0)	500 (31)	494 (31)	459 (29)	487 (31)	445 (28)
Obese (≥30.0)	519 (33)	494 (31)	486 (31)	491 (31)	524 (33)

Abbreviations: BMI, body mass index (calculated as weight in kilograms divided by height in meters squared); FPL, federal poverty level.

^a^ Missing data ranged from 0.0%-0.2%.

### Beverage Calories

**Figure 3.**
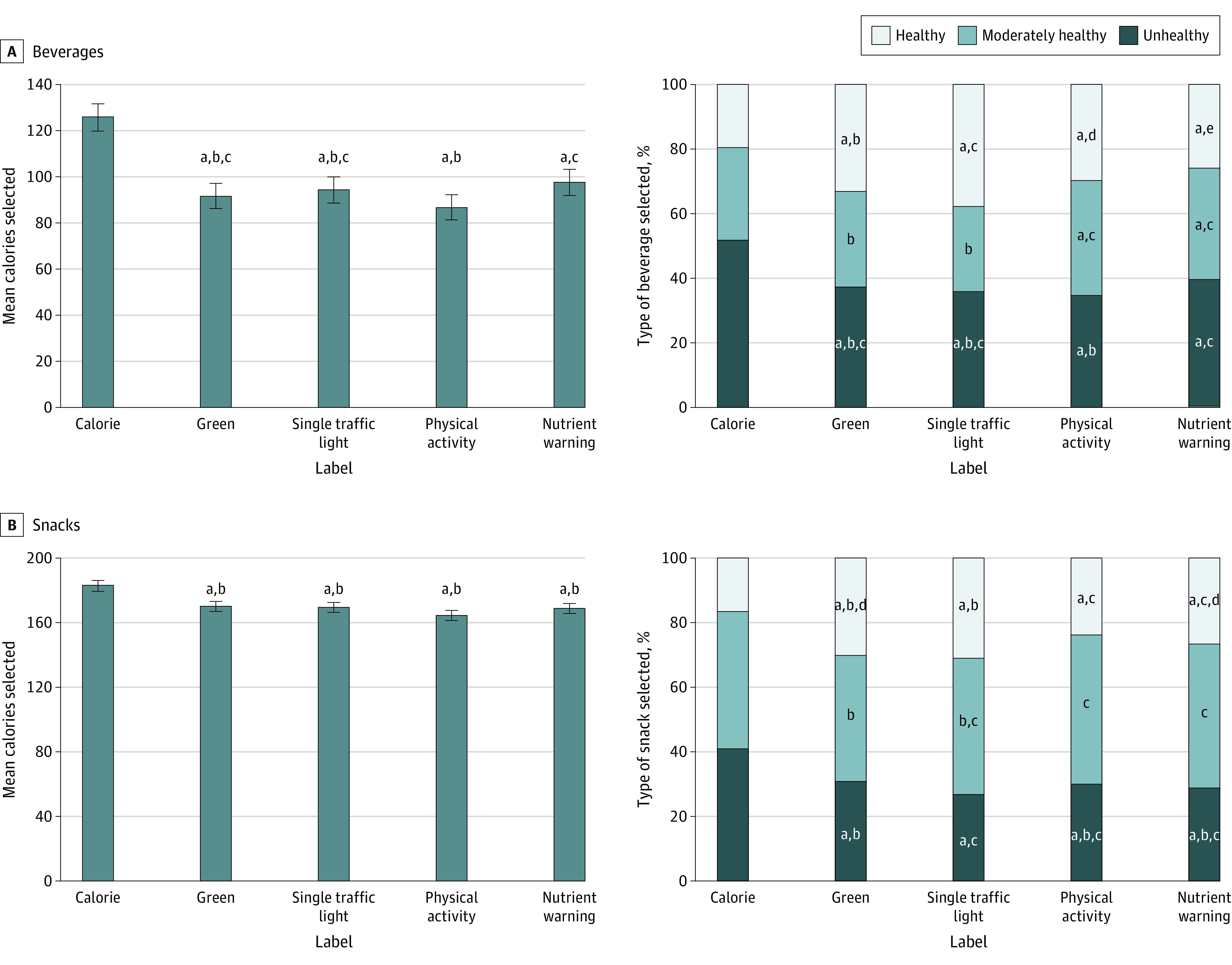
Effect of Interpretative Front-of-Package Labels on Beverage and Snack Selections Error bars indicate 95% CIs. ^a-e^Means and percentages that do not share a superscript letter (including the absence of a letter) with one another are statistically different (Holm-Bonferroni–corrected *P* < .05) from one another in their effect on the outcomes (4 tests per outcome to compare each interpretative label to control and 6 tests per outcome to compare the 4 interpretative labels with one another).

Participants assigned to the control arm selected a mean (SD) of 125.8 (116.8) calories from beverages (Figure 3 and eTable 5 in Supplement 2). Participants exposed to the green (ADE, −34.2; 95% CI, −42.2 to −26.1), traffic light (ADE, −31.5; 95% CI, −39.5 to −23.4), physical activity (ADE, −39.0; 95% CI, −47.0 to −31.1), or nutrient warning (ADE, −28.2; 95% CI, −36.2 to −20.2) labels selected fewer beverage calories than participants in the control arm (all *P* < .001). These ADEs represent reductions of approximately 22% to 31% compared with the control arm. There were no differences among the interpretative labeling arms in their effects on beverage calories except that the physical activity labels led to a larger reduction than the nutrient warnings (eTable 6 in Supplement 2). The effects of the interpretative labeling systems on beverage calories did not differ by education level (χ^2^_8_ = 3.76; *P* = .88; eTable 7 in Supplement 2).

### Snack Calories

In the control arm, participants selected a mean (SD) of 182.8 (69.2) calories from snacks. Participants exposed to the green (ADE, −12.7; 95% CI, −17.3 to −8.2), traffic light (ADE, −13.7; 95% CI, −18.2 to −9.1), physical activity (ADE, −18.5; 95% CI, −23.1 to −13.9), or nutrient warning (ADE, −14.2; 95% CI, −18.8 to −9.6) labels selected fewer snack calories than participants in the control arm (all *P* < .001). These ADEs represent reductions of approximately 7% to 10% compared with the control arm. The 4 interpretative labeling systems did not differ from one another in their effects on snack calories (eTable 6 in Supplement 2). The effects of the interpretative labeling systems on snack calories did not differ by education level (*F*^4,7935^ = 1.88; *P* = .11; eTable 7 in Supplement 2).

### Secondary Nutritional Outcomes

Compared with control, participants exposed to the green labels were less likely to select an unhealthy beverage (ADE, −14.5 percentage points; 95% CI, −17.9 to −11.0 percentage points) and more likely to select a healthy beverage (ADE, 13.6 percentage points; 95% CI, 10.6-16.6 percentage points) (Figure 3). Effects were similar for the traffic light, physical activity, and nutrient warning labels. The 4 interpretative labeling systems also generally led to lower sugar and sodium selected from beverages compared with control (eTables 5 and 6 in Supplement 2). None of the interpretative labeling systems consistently outperformed the others on secondary nutritional outcomes for beverages.

Similar to the results for beverage selections, the 4 interpretative labeling systems led to lower likelihood of selecting an unhealthy snack and higher likelihood of selecting a healthy snack. Participants in the interpretative arms also selected fewer grams of sugar and fewer grams of saturated fat from snacks than the control arm (eTable 6 in Supplement 2). Only the physical activity labels led to reductions in sodium selected from snacks (ADE, −7.6 mg; 95% CI, −12.5 to −2.6 mg). None of the interpretative labeling systems consistently outperformed the others on secondary nutritional outcomes for snacks.

### Label Reactions

The traffic light, physical activity, and nutrient warning labels elicited stronger label reactions than the calorie labels, including higher ratings on attention, thinking about health effects, negative emotions, perceived discouragement from buying unhealthy products, and perceived encouragement to buy healthy products (Table 2 and eTable 8 in Supplement 2). By contrast, the green labels generally led to weaker label reactions than the calorie labels, including lower ratings than the calorie labels for attention, thinking about harms, negative emotions, and perceived discouragement.

**Table 2.** Effects of Interpretative Front-of-Package Labeling Systems on Perceptions and Label Reactions (N = 7945)^a^

Outcome (response coding)	Control, mean (SD) (n = 1593)	Green labels (n = 1587)		Single traffic light labels (n = 1588)		Physical activity labels (n = 1590)		Nutrient warning labels (n = 1587)	
		Mean (SD)	Corrected *P *value for ADE	Mean (SD)	Corrected *P *value for ADE	Mean (SD)	Diff vs control, corrected *P *value	Mean (SD)	Corrected *P *value for ADE
Label reactions									
Noticed label (0/1), No. (%)	538 (34)	692 (44)	<.001	711 (45)	<.001	772 (49)	<.001	960 (61)	<.001
Attention to label (1-5)	3.3 (1.2)	3.2 (1.2)	.02	3.4 (1.2)	<.001	3.5 (1.2)	<.001	3.5 (1.2)	<.001
Thinking about health effects (1-5)	3.2 (1.2)	3.0 (1.3)	.003	3.3 (1.2)	<.001	3.4 (1.3)	<.001	3.5 (1.2)	<.001
Negative emotions (1-5)	1.7 (0.9)	1.5 (0.8)	<.001	1.8 (0.9)	.01	1.9 (1.0)	<.001	2.0 (1.0)	<.001
Discouragement from buying unhealthy foods (1-5)	2.7 (1.3)	2.5 (1.3)	<.001	2.9 (1.3)	.001	3.1 (1.3)	<.001	3.2 (1.3)	<.001
Encouragement to buy healthy foods (1-5)	3.0 (1.3)	3.0 (1.3)	.85	3.2 (1.3)	<.001	3.3 (1.3)	<.001	3.3 (1.3)	<.001
Label influenced beverage choice (0/1), No. (%)	658 (41)	578 (36)	.009	698 (44)	.13	805 (51)	<.001	915 (58)	<.001
Label influenced snack choice (0/1), No. (%)	563 (35)	522 (33)	.14	669 (42)	<.001	785 (49)	<.001	872 (55)	<.001
Message reactance (1-5)	1.9 (1.0)	2.0 (1.1)	.001	2.2 (1.1)	<.001	2.0 (1.1)	.003	2.1 (1.1)	<.001
Healthfulness perceptions									
Healthfulness of healthy beverage (1-5)	3.8 (0.9)	4.0 (0.9)	<.001	4.0 (1.0)	<.001	4.0 (1.0)	<.001	4.0 (1.0)	<.001
Healthfulness of moderately healthy beverage (1-5)	3.7 (1.0)	3.5 (1.0)	<.001	3.3 (1.0)	<.001	3.6 (1.0)	.002	3.7 (1.0)	.43
Healthfulness of unhealthy beverage (1-5)	3.4 (1.1)	3.2 (1.1)	<.001	2.7 (1.2)	<.001	3.0 (1.1)	<.001	2.8 (1.2)	<.001
Healthfulness of healthy snack (1-5)	3.3 (1.0)	3.4 (1.0)	<.001	3.6 (1.1)	<.001	2.9 (1.0)	<.001	3.1 (1.0)	<.001
Healthfulness of moderately healthy snack (1-5)	2.7 (1.0)	2.7 (1.0)	.23	2.7 (1.0)	.52	2.6 (1.0)	.06	2.4 (1.0)	<.001
Healthfulness of unhealthy snack (1-5)	2.3 (1.0)	2.3 (1.0)	.90	2.0 (1.0)	<.001	2.2 (1.0)	.06	2.0 (1.0)	<.001
Stigma									
Perceived personal stigma (1-5)	1.9 (1.2)	1.8 (1.1)	.36	2.1 (1.2)	<.001	2.0 (1.2)	.01	2.1 (1.2)	<.001
Perceived obesity stigma (1-5)	2.1 (1.2)	1.9 (1.1)	<.001	2.3 (1.2)	<.001	2.2 (1.2)	.11	2.4 (1.2)	<.001
Disgust toward people with obesity (1-5)	2.0 (1.2)	1.9 (1.2)	.69	2.0 (1.1)	.81	1.9 (1.2)	>.99	2.0 (1.2)	.70
Positive label perceptions									
Learned something new from the label (1-5)	2.7 (1.2)	2.8 (1.3)	.009	3.1 (1.2)	<.001	3.5 (1.2)	<.001	3.2 (1.2)	<.001
Trust information in the label (1-5)	3.8 (1.0)	3.5 (0.9)	<.001	3.6 (1.0)	<.001	3.7 (0.9)	.03	3.8 (0.9)	.02
Perceptions of control over healthy eating (0/1), No. (%)	742 (47)	659 (42)	.009	771 (49)	.27	865 (54)	<.001	916 (58)	<.001
Used label to help choose beverage (1-5)	2.6 (1.3)	2.4 (1.3)	<.001	2.6 (1.3)	.41	2.8 (1.4)	<.001	3.0 (1.4)	<.001
Used label to help choose snack (1-5)	2.4 (1.3)	2.4 (1.3)	.24	2.6 (1.3)	<.001	2.8 (1.4)	<.001	3.0 (1.4)	<.001

Abbreviation: ADE, average differential effect.

^a^ This table shows means and SDs of psychological secondary outcomes by trial arm and whether outcomes for the interpretative front-of-package labeling arms differed from the control arm (corrected *P* < .05). The Holm-Bonferroni method was used to correct *P* values for multiple comparisons, considering 4 tests per outcome (each interpretative label vs control). Average differential effects of the interpretative labeling systems (vs control) are shown in eTable 8 in Supplement 2.

### Healthfulness Perceptions

Compared with control, the green labels led to higher perceived healthfulness of the healthy beverage (ADE, 0.23; 95% CI, 0.16-0.30) and lower perceived healthfulness of the unhealthy beverage (ADE, −0.14; 95% CI, −0.21 to −0.08). The traffic light, physical activity, and nutrient warning labels had similar effects on healthfulness perceptions of beverages (eTable 8 in Supplement 2). The healthfulness perceptions of snacks were not as consistent; for example, the physical activity and nutrient warning labels led to unexpectedly lower perceived healthfulness of healthy snacks compared with control (eTable 8 in Supplement 2).

### Stigma

Perceived personal stigma ratings were relatively low for the calorie label (mean [SD], 1.9 [1.2] on the 1-5 scale). Participants rated the traffic light (ADE, 0.16; 95% CI, 0.09-0.23), physical activity (ADE, 0.10; 95% CI, 0.03-0.17), and nutrient warning (ADE, 0.19; 95% CI, 0.12-0.26) labels, but not the green label (ADE, −0.03; 95% CI, −0.10 to 0.04), as more personally stigmatizing than the calorie label. Compared with the calorie label, the traffic light (ADE, 0.13; 95% CI, 0.06-0.19) and nutrient warning (ADE, 0.19; 95% CI, 0.13-0.26) labels led to higher perceptions that the labels stigmatized people with obesity, while the green label lowered this perception (ADE, −0.21; 95% CI, −0.27 to −0.14). None of the 4 interpretative labels led to higher disgust toward people with obesity. The effects of the interpretative labels on stigma outcomes were not moderated by obesity status (eTable 9 in Supplement 2).

### Label Perceptions

The nutrient warning and physical activity labels generally elicited the most favorable label perceptions. For example, the nutrient warnings received the highest ratings on trustworthiness, while the physical activity labels received the highest ratings on participants’ perceptions that they learned something new from the labels (eTable 8 in Supplement 2). The green label was generally perceived less favorably than the other 3 interpretative labeling systems.

## Discussion

In this large randomized clinical trial, exposure to green “choose often,” single traffic light, physical activity, or nutrient warning front-of-package labeling systems led participants to select beverages and snacks with fewer calories compared with exposure to calorie labels alone. These 4 interpretative labeling systems also reduced selection of sugar from beverages and sugar and saturated fat from snacks. These results reinforce prior studies indicating that interpretative food labels are more effective at promoting healthier food selection than numeric labels.^11,17,43,44,45^

The 4 interpretative labeling systems led to similar effects on beverage and snack calories selected regardless of participants’ education level. To our knowledge, this is among the largest randomized trials to examine whether the effects of interpretative food labels differ by education level.^17,30,31,46,47,48,49,50^ The present results suggest that interpretative food labels would be unlikely to exacerbate education-based disparities in dietary quality, an encouraging finding given that Chile’s nutrient warnings may have yielded larger benefits among more highly educated households.^51^

In general, the 4 interpretative labeling systems performed similarly to one another in their effects on calories, sugar, saturated fat, and sodium selected from beverages and snacks. By contrast, the 4 interpretative labeling systems differed in how they affected psychological outcomes. For example, the nutrient warning labels elicited the most attention, thinking about health effects, and negative emotions, followed by the physical activity and traffic light labels. The green labels elicited the least attention, thinking about health effects, and emotions. Because these reactions are associated with behavior change,^34,36,52,53,54^ the present results suggest that traffic light labels, physical activity labels, and nutrient warning labels might be more promising than green labels for spurring long-term behavior change. These results align with research finding that endorsement labels like the green “choose often” label used in this study are generally less effective than labels that explicitly discourage unhealthy foods.^11,17,44,55^

This study is among the first to examine the effects of interpretative food labeling systems on stigma,^13,37^ an important contribution given that stigma toward people with obesity is pervasive and harmful to mental and physical health.^56,57,58^ In the present study, none of the labeling systems elicited high absolute levels of stigma (mean ratings were all <2.5 on the 1-5 scales), though we did find that some of the interpretative labeling systems elicited slightly more stigma than the calorie labels. Importantly, none of the interpretative labels led to higher feelings of disgust toward people with obesity,^37^ suggesting that interpretative labeling systems can be designed to encourage healthier purchases with minimal or no increases in stigma.

Together, these results suggest that policymakers have a variety of promising policy options when selecting an interpretative front-of-package labeling system to encourage healthier purchases. Policymakers’ choice of system might depend on how they weigh different criteria (eg, whether high trustworthiness or low stigma is more important). Policymakers might also consider how each system could further other goals, such as whether certain systems are more likely to elicit beneficial product reformulation^59,60^ or improve consumer understanding of product healthfulness.^11,61^ Finally, policymakers might consider how different labeling systems could undergird other regulations, such as taxes, marketing restrictions, or school nutrition standards.^11^

### Strengths and Limitations

This study had limitations. First, we measured hypothetical selections, although we incentivized realistic behavior. Second, participants had only brief exposure to labels in the context of an online vending machine experiment. Future research should explore the effects of repeated, in-person exposure to labels and examine effects in more contexts (eg, when selecting groceries). Third, we did not study some of the labeling systems in use internationally, such as the Nutri-Score, Heath Star Rating, or multiple traffic light labeling systems, and we did not examine other important aspects of label design, such as whether labels include government attritubtion.^62,63,64^ Fourth, of the labels we tested, only nutrient warnings have been broadly implemented. Fifth, some of the labeling systems we tested used different underlying nutrition criteria, so we cannot separate the effects of label design vs the criteria for applying the labels. However, this study design reflects real-world differences in how labels would be implemented. Strengths of this study included the randomized design, realistic stimuli, and large sample that enabled us to compare different label designs with one another and examine whether education level moderated label effects on product selections.

## Conclusions

In this randomized clinical trial of front-of-package food labeling systems, all 4 interpretative labeling systems reduced calories selected from beverages and from snacks compared with calorie labels, including among people with different education levels. Single traffic light, physical activity, and nutrient warnings showed more promise for spurring long-term behavior change than green labels.

## Notes

Supplement 1.
Trial Protocol

Supplement 2.
eMethods.
**eFigure.** Digital posters used in the trial to explain labeling systems to participants in the green label arm (panel A), single traffic light labels arm (panel B), physical activity labels arm (panel C), and the nutrient warning labels arm (panel D) **eTable 1.** Characteristics of products offered in the trial **eTable 2. **Secondary psychological outcomes measured in the randomized trial **eTable 3.** Survey measures used in the trial **eTable 4.** Comparison of characteristics of the study sample (n=7,945 U.S. adults) to national estimates **eTable 5.** Calories, nutrients, and type of items selected in beverage and snack selection tasks by trial arm, n=7,945 **eTable 6.** Effects of interpretative front-of-package labeling systems on beverage and snack selections, n=7,945 **eTable 7.** Effects of interpretative front-of-package labeling systems on beverage calories and snack calories selected, by education level **eTable 8. **Effects of front-of-package labeling systems on perceptions and label reactions, n=7,945 **eTable 9.** Effects of interpretative front-of-package labeling systems on stigma outcomes, by obesity status
eReferences

Supplement 3.
Data Sharing Statement
