# Supplementary material for: Effects of 4 Interpretive Front-of-Package Labeling Systems on Hypothetical Beverage and Snack Selections: A Randomized Clinical Trial
Source: JAMA Netw Open. 2023 Sep 13;6(9):e2333515. doi: 10.1001/jamanetworkopen.2023.33515 (PMC10500374; doi:10.1001/jamanetworkopen.2023.33515)
Supplement: Supplement 1. — Trial Protocol [file jamanetwopen-e2333515-s001.pdf]

Effects of Four Interpretive Front-of-Package Labeling Systems on Beverage and Snack Selections: A Randomized Clinical Trial Trial Protocol and Analysis Plan

Notes

Per journal instructions, this document contains the trial protocol and analysis plan as it was submitted to the University of Pennsylvania Institutional Review Board. We additionally pre-registered the analysis plan through AsPredicted (https://aspredicted.org/XK9_4M4). Deviations from this plan are detailed and justified in the main text.

Protocol

Objectives

Overall objectives

1. Test the effect of front-of-package (FOP) nutrition labels on consumers' hypothetical purchases, perceptions, and knowledge.
2. Test whether effects differ by type of FOP nutrition label (control, green light, traffic lights (red, yellow, green), physical activity labels, or "High in" warnings).

Background

Poor dietary habits are a significant public health problem. Unhealthy dietary habits are associated with obesity, cardiovascular disease, type 2 diabetes, and certain cancers. (1). To comprehensively address these chronic diseases, interventions need to focus on calorie reduction and changing specific dietary habits. For example, potato chips and sweetened beverages are two foods with the strongest links to long-term weight gain, suggesting a need to focus on decreasing their consumption, while vegetables, whole grains, fruits, nuts, and yogurt are protective against weight gain (2). This project aims to determine which nutrition messages are best able to encourage lower calorie and higher nutritional quality food choices.

Additionally, governments and companies are increasingly interested in using front-of-package (FOP) nutrition labels to provide consumers with accessible, easy-to-understand nutrition information (3). Questions remain about how to design FOP nutrition labels to maximize their effectiveness at informing consumers and promoting healthier food and beverage purchases. For example, some labeling schemes such as Smart Choices and Guiding Stars use "endorsement logos" to signal when products meet nutritional standards. Other schemes, such as those adopted in Chile, Brazil, and Mexico, use warning labels to alert consumers when products are high in unhealthy nutrients like sodium, sugar, or saturated fat. Traffic lights combine these approaches, highlighting healthier items with green labels moderately unhealthy items with yellow labels, and less healthy items with red labels. These approaches could affect consumer understanding and purchase decisions (3). Limited research, however, has compared these approaches head-to-head. The objective of this study is to evaluate the impact of 5 types of FOP nutrition labels on calories from snacks and beverage purchases and probability of selecting a healthy, moderately unhealthy, and unhealthy item.

1. Mozaffarian D. Dietary and policy priorities for cardiovascular disease, diabetes, and obesity: a comprehensive review. Circulation. 2016;133(2):187-225.
2. Mozaffarian D, Hao T, Rimm EB, Willett WC, Hu FB. Changes in diet and lifestyle and long-term weight gain in women and men. N Engl J Med. 2011;364(25):2392-2404.
3. Roberto CA, Ng SW, Ganderats-Fuentes M, et al. The Influence of Front-of-Package Nutrition Labeling on Consumer Behavior and Product Reformulation. *Annu Rev Nutr*. Published online September 20, 2021. doi:10.1146/annurev-nutr-111120-094932

Study Design

Design

This project involves an online randomized experiment. We are working with an external survey company that will recruit 8,000 participants to complete an online survey in which they will be randomized to one of the five different label conditions: 1) calorie labels (control condition); 2) single green traffic light labels (on healthy foods); 3) multiple traffic light labels (red traffic light labels applied to least healthy foods, yellow applied to moderately unhealthy foods, and green on healthy foods); 4) physical activity labels (calorie content displayed in terms of physical activity equivalents); 5) "High in" warnings (applied to all products exceeding thresholds for calories, saturated fat, sugar, or sodium content). Participants will be instructed to select products to purchase in a mock vending machine. After purchasing their products, they will be asked to answer questions about their perceptions of the different labels.

Study duration
- Estimated length of enrollment and study completion: 2 months
- The entire survey and purchasing task are estimated to take 12-15 minutes.
- Project date: we aim to launch once we receive IRB approval and finalize our survey with the survey company

Characteristics of the Study Population

Target population
We are working with a survey company to recruit a nationally convenience sample of 8,000 adults.

Subjects enrolled by Penn Researchers
8,000

Subjects enrolled by Collaborating Researchers
0

Vulnerable Populations

Children Form
Pregnant women (if the study procedures may affect the condition of the pregnant woman or fetus) Form
Fetuses and/or Neonates Form
Prisoners Form
Other
x **None of the above populations are included in the research study**
The following documents are currently attached to this item:
There are no documents attached for this item.

Subject recruitment
We will work with the survey company CloudResearch to recruit participants. We aim to get a national sample of 8,000 adults 18+ that will match the US population parameters (gender, race/ethnicity) based on the US Census data. For our national sample, we aim to get a sample that is split 50/50 in low education (defined as some college, high school, less) and high education (defined as associates, BA, more) across the age groups.

CloudResearch will use Prime Panels to recruit participants. The market research platforms that make up Prime Panels have their own participant pools (opt-in panels). Participants on these panels are profiled on hundreds of variables. Invitations to participate in research studies are sent via email and dashboards to specific participants, based on their demographic characteristics. Prime Panel methodology via CloudResearch allows us to recruit our specific and representative sample for this study.

Will the recruitment plan propose to use any Penn media services (communications, marketing, etc.) for outreach via social media avenues (examples include: Facebook, Twitter, blogging, texting, etc.) or does the study team plan to directly use social media to recruit for the research?
No
The following documents are currently attached to this item:
There are no documents attached for this item.

Subject compensation*
Will subjects be financially compensated for their participation?
Yes
The following documents are currently attached to this item:
There are no documents attached for this item.

If there is subject compensation, provide the schedule for compensation per study visit or session and total amount for entire participation, either as text or separate document
The survey company will provide a small compensation to the research participants. The final details will be determined by the survey company. Recruitment materials will include information on compensation amount and type (e.g., gift cards, points redeemable for prizes).

To encourage participants to select items in the vending machine choice tasks that they are actually interested in purchasing, the survey will instruct participants that some respondents will be selected at random for a “bonus” in which they have one of their product selections delivered to them. In reality, at the end of the study, we will debrief participants on the study purpose and inform them that all participants who won the “bonus” will be compensated with an electronic gift card with a $2.50 balance rather than being mailed the product.

Study Procedures

Suicidal Ideation and Behavior
Does this research qualify as a clinical investigation that will utilize a test article (ie- drug or biological) which may carry a potential for central nervous system (CNS) effect(s)?
No

Procedures
This study involves one online randomized experiment. 8,000 participants will be recruited via the survey company CloudResearch and will be asked to participate in a study. After consent is obtained, participants will be randomized to one of five label conditions: 1) calorie labels (control condition); 2) single green traffic light labels (on healthy foods); 3) multiple traffic light labels (red traffic light labels applied to least healthy foods, yellow applied to moderately unhealthy foods, and green on healthy foods); 4) physical activity labels (calorie content displayed in terms of physical activity equivalents); 5) “High in” warnings (applied to all products exceeding thresholds for calories, saturated fat, sugar, or sodium content).

Once in the study, participants will be instructed to select products to purchase in a mock online vending machine. They will complete two vending machine choice tasks, one for snack foods, and one for beverages. In each task, products will be shown in a random arrangement of the products with the labels for the participants’ assigned group shown on the product’s packaging as well as with an enlarged call-out of the product’s label. For each task, participants will be asked to select at least 1 item they would like to purchase. To incentivize truthful responding, participants will be informed that they will be selected at random for a “bonus” in which they will have their vending machine selection delivered to an address of their choosing. In reality at the end of the study, we will debrief participants on the study purpose and inform them that all participants who are randomly selected to receive the bonus will receive an electronic gift card worth $2.50. This very minor deception enables us to incentivize participants to behave as they normally would when choosing vending machine items and to minimize potential social desirability biases introduced by participating in a study. Participants will then be asked to complete a survey with questions about their perceptions of the FOP labels.

They will also provide demographic information about themselves. After data are collected, participants will be excluded if they have a 1) repeat IP address; or 2) they complete the survey in 1/3 median survey completion time.

The following documents are currently attached to this item:
There are no documents attached for this item.

Deception
Does your project use deception?
Yes

Participants will be told to select items they wish to purchase from the mock vending machines and that some participants (chosen at random) will receive their selected item delivered to them. At the end of the study, we will debrief participants on the study purpose and inform them that the randomly selected participants will receive an electronic gift card with a value of $2.50 instead of the item. Using this very minor deception allows us to incentivize participants to behave as they normally would when choosing products from a vending machine and to minimize potential social desirability bias introduced by participating in a study, while avoiding having to collect their addresses to ship items. Moreover, we expect that most participants will prefer to receive a gift card over their selected products, as the gift card can be used to purchase any items, including beverages or snacks.

After completing the survey, participants will receive a debriefing form explaining this minor deception, and they will have the opportunity to check a box if they wish to exclude their data from use in our study (see Debriefing Materials attached).

International Research
Are you conducting research outside of the United States?
No

Analysis Plan
Overview
Participant characteristics and purchasing behaviors will be descriptively summarized overall and by intervention arm prior to analysis. Means and standard deviations will be used to characterize continuous variables (such as age and calories purchased), and frequencies and percentages will be used to describe categorical variables (such as gender, educational attainment, and nutritional quality of items purchased). Medians and interquartile ranges will be reported for continuous variables that exhibit skewness.

All analyses will be stratified by snacks or beverages, and all outcomes will be analyzed at the level of the item purchased. Participants are required to choose a beverage and a snack in order to complete the task, so we do not expect substantial missing data.

Main Effects
Calories purchased. Analyses will examine the impact of labeling arm on calories purchased in the vending machine tasks. For the snack choice task, we will use ordinary least squares (OLS) regression, regressing calories on a set of indicator variables for labeling arm. For the beverage choice task, we anticipate calories purchased will be zero-inflated, so plan to use a two-part model with logistic regression to examine probability of purchasing any calories and OLS regression to examine amount of calories purchased conditional on having purchased any calories. For both beverages and snacks, we will estimate marginal effects of each intervention label (vs. control). Next, we will compare the effects of each intervention label (vs. control) to one another using Chi-Squared tests. We will use the Holm-Bonferroni method to account for multiple comparisons.

Secondary outcomes. Using a similar approach as the primary analyses, we will additionally examine the impact of the interpretive FOP labels (vs. the control condition) on the following secondary outcomes: likelihood of selecting a healthy, moderately healthy, or unhealthy item; mean nutrients selected in the choice tasks (saturated fat, sodium, sugar); noticing of labels; perceptions that labels influenced product selection; trust in labels, perceived product healthfulness; reactance to labels; attention to labels; thinking about health effects; perceived message effectiveness; negative emotional reactions; perceived control in response to labels, and perceived personal and obesity stigma. We will use linear models for continuous outcomes (or two-part models if outcomes are zero-inflated), logistic models for binary outcomes, and ordered logistic models for ordered categorical outcomes (or multinomial logit if data show evidence of lack of proportionality). We will adjust for multiple comparisons using the Holm-Bonferroni approach, considering each outcome its own family of tests.

Moderation
Analyses will also examine whether the effect of intervention labels on calories purchased differed by educational attainment. These moderation analyses regressed outcomes on labeling arm, the potential effect modifier (i.e., educational attainment level), and their interaction. For the separate beverage and snack models, we will examine the joint significance of the interaction terms between education level and the labeling arms. If our main effects analyses (described above) reveal limited differences between the 4 interpretive labeling label arms, we will consider collapsing across interpretive labeling arms (i.e., using a single indicator variable for exposure to interpretive labels vs. control) for moderation analyses. The presence of an interaction will be evaluated by the two-sided test of the null hypothesis that the interaction coefficient is 0. Tests of interactions will be conducted at the 0.05 level.
