# Supplementary material for: Effects of 4 Interpretive Front-of-Package Labeling Systems on Hypothetical Beverage and Snack Selections: A Randomized Clinical Trial
Source: JAMA Netw Open. 2023 Sep 13;6(9):e2333515. doi: 10.1001/jamanetworkopen.2023.33515 (PMC10500374; doi:10.1001/jamanetworkopen.2023.33515)
Supplement: Supplement 2. — eMethods. eFigure. Digital posters used in the trial to explain labeling systems to participants in the green label arm (panel A), single traffic light labels arm (panel B), physical activity labels arm (panel C), and the nutrient warning labels arm (panel D) eTable 1. Characteristics of products offered in the trial eTable 2. Secondary psychological outcomes measured in the randomized trial eTable 3. Survey measures used in the trial eTable 4. Comparison of characteristics of the study sample (n=7,945 U.S. adults) to national estimates eTable 5. Calories, nutrients, and type of items selected in beverage and snack selection tasks by trial arm, n=7,945 eTable 6. Effects of interpretative front-of-package labeling systems on beverage and snack selections, n=7,945 eTable 7. Effects of interpretative front-of-package labeling systems on beverage calories and snack calories selected, by education level eTable 8. Effects of front-of-package labeling systems on perceptions and label reactions, n=7,945 eTable 9. Effects of interpretative front-of-package labeling systems on stigma outcomes, by obesity status eReferences [file jamanetwopen-e2333515-s002.pdf]

# Supplemental Online Content

Grummon AH, Gibson LA, Musicus AA, Stephens-Shields AJ, Hua SV, Roberto CA. Effects of 4 interpretive front-of-package labeling systems on hypothetical beverage and snack selections: a randomized clinical trial. *JAMA Netw Open*. 2023;6(9):e2333515. doi:10.1001/jamanetworkopen.2023.33515

**eMethods.**
**eFigure.** Digital posters used in the trial to explain labeling systems to participants in the green label arm (panel A), single traffic light labels arm (panel B), physical activity labels arm (panel C), and the nutrient warning labels arm (panel D)
**eTable 1.** Characteristics of products offered in the trial
**eTable 2.** Secondary psychological outcomes measured in the randomized trial
**eTable 3.** Survey measures used in the trial
**eTable 4.** Comparison of characteristics of the study sample (n=7,945 U.S. adults) to national estimates
**eTable 5.** Calories, nutrients, and type of items selected in beverage and snack selection tasks by trial arm, n=7,945
**eTable 6.** Effects of interpretative front-of-package labeling systems on beverage and snack selections, n=7,945
**eTable 7.** Effects of interpretative front-of-package labeling systems on beverage calories and snack calories selected, by education level
**eTable 8.** Effects of front-of-package labeling systems on perceptions and label reactions, n=7,945
**eTable 9.** Effects of interpretative front-of-package labeling systems on stigma outcomes, by obesity status
**eReferences**

This supplemental material has been provided by the authors to give readers additional information about their work.

© 2023 Grummon AH et al. *JAMA Netw Open.*

## eMethods. Supplementary methodological details

**Detailed Descriptions of the Trial Arms**

Calorie labels were displayed on all products in all trial arms. The survey software randomized participants to one of five trial arms using a simple allocation ratio: 1) Control (calorie labels only on all products); 2) Green labels (green "choose often" labels added to healthier products with no additional labels on products not meeting healthfulness criteria); 3) Single traffic light labels (hereafter "traffic light labels;" green "choose often," yellow "choose sometimes," and red "choose rarely" labels added to healthy, moderately healthy, and unhealthy products, respectively); 4) Physical activity labels (labels showing calorie content expressed as minutes of physical activity required to burn those calories added to all products); or 5) Nutrient warning labels ("high in" nutrient warning labels added to products exceeding thresholds for sugar, sodium, saturated fat, or calories). Interpretative labels were added to the calorie labels in the treatment arms, as described below. For the green labels, single traffic light labels, and physical activity labels, we applied the labels based on the criteria we used in a recent field experiment of these labeling systems to enable comparison to those results (Gibson et al., 2022). For the nutrient warnings, we used criteria from the 2018 thresholds of the Chilean Law of Food Labeling and Advertising (Corvalán et al., 2013). The trial arms therefore reflect how these labeling systems have been implemented in the real world.

**All conditions.** Calorie labels were displayed on the front of all products. The calorie labels depicted the number of calories in the beverage or snack (i.e., calories per product).

**Control.** The control condition showed calorie labels only.

**Green labels**. Green "choose often" labels were added to the front of healthy products; no labels were added to moderately healthy or unhealthy products. Beverages were considered healthy if they had ≤ 5g sugar per 12 oz. Snacks were considered healthy if they had ≤ 250 calories, ≤ 5g total fat (except for nuts and seeds), ≤ 1 g saturated fat (except for nuts and seeds), and ≤ 18 g of sugar (except for fruit, nuts, and seeds with no added sugar) per package. These criteria were based on nutrition standards used in the City of Philadelphia to highlight healthier options or right-sized portions in city vending machines (Gibson et al., 2022).

**Single traffic light labels.** Green "choose often" labels were added to the front of healthy products, yellow "choose sometimes" labels were added to moderately healthy products, and red "choose rarely" labels were added to unhealthy products. The criteria for being considered healthy are described above. Beverages were considered moderately healthy if they contained 6-12g sugar per 12 oz or if they contained artificial sweeteners. Beverages were considered unhealthy if they contained ≥ 12g sugar per 12 oz. Snacks were considered moderately healthy if they contained 250–350 calories, 5–9g total fat, 1–3g saturated fat (except for nuts and seeds), or >18g sugar (including only fruit, nuts, and seeds with added sugar). Snacks were considered unhealthy if they contained >350 calories, >9g total fat, >3g saturated fat (except for nuts and seeds), or >18g sugar. These criteria were based on nutrition standards used in the City of Philadelphia to highlight healthier options or right-sized portions in city vending machines (Gibson et al., 2022).

**Physical activity labels**. Physical activity labels expressing calorie content in number of minutes needed to burn those calories were added to the front of all products. Physical activity calorie equivalents were estimated as 0.45 minutes per calorie.

**Nutrient warning labels**. Nutrient warning labels were added to products "high in" calories, sugar, sodium, or saturated fat. We defined "high in" using the 2018 thresholds of the Chilean Law of Food Labeling and Advertising (Corvalán et al., 2013). For foods, these cutoffs are: >300kcal per 100g, >15g sugar per 100g, >5g saturated fat per 100g and >500mg sodium per 100g of food. For beverages, these cutoffs are: >80kcal per 100mL, >5g sugar per 100mL, >3g saturated fat per 100mL and >100mg sodium per 100mL of beverage.

**Details on Power Calculations**

We powered the study to detect an effect of each interpretive labeling system (vs. control) on beverage calories selected, which we expected would show higher variability compared to snack calories due to 7 of 16 beverages having 0 calories (zero inflation) and thus require a larger sample. Based on our sales data from the field experiment (Gibson et al., 2022), we assumed 50% of participants would select a zero-calorie beverage and a standard deviation

of 66 calories among those with non-zero calories. Using these parameters and assuming alpha=0.0125 to account for 4 tests (each interpretative label vs. control), the target total sample size of 8,000 (1,600 per labeling arm) would yield 80% power to detect an effect of each interpretative label vs. control on calories selected from beverages of *d*=0.167 or larger, similar to the effect of sugary drink labels in a meta-analysis(Grummon & Hall, 2020) and corresponding to a difference of 11 kcal. This sample would yield 80% power to detect an interaction between the front-of-package label condition and education of *d*=0.28 (18.5 kcal) assuming alpha=0.05.

**eFigure. Digital posters used in the trial to explain labeling systems to participants in the green label arm (panel A), single traffic light labels arm (panel B), physical activity labels arm (panel C), and the nutrient warning labels arm (panel D)**

**(A) Green label arm**

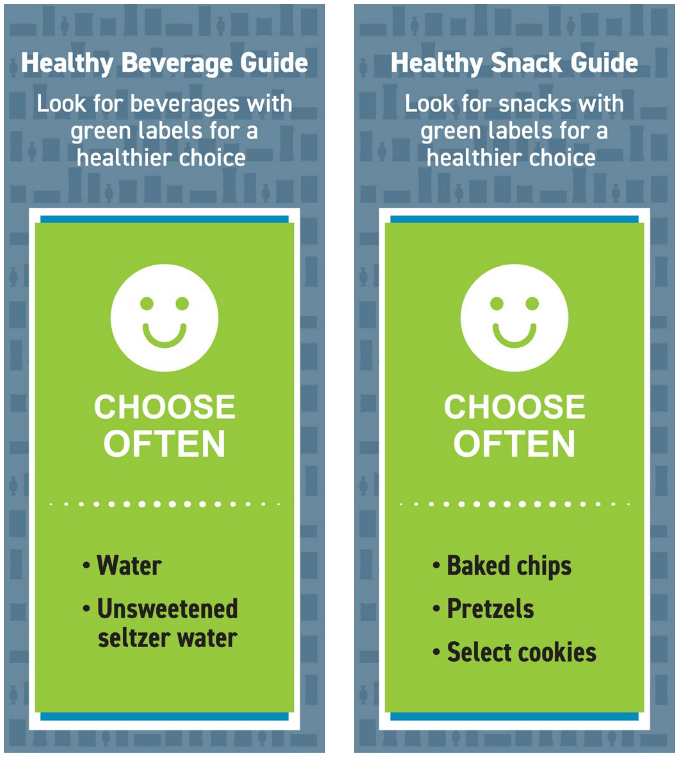


**(B) Single traffic light labels arm**

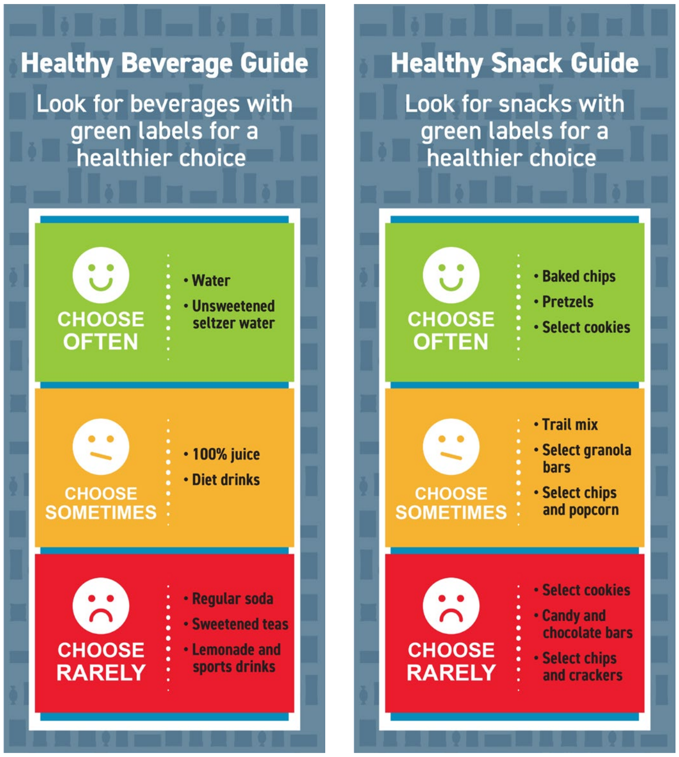


**(C) Physical activity labels arm**

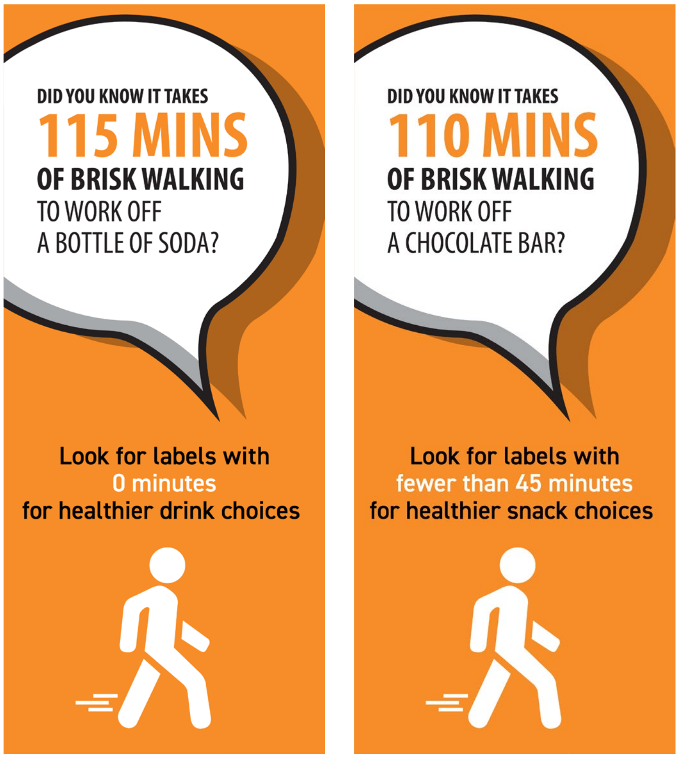


**(D) Nutrient warning labels arm**

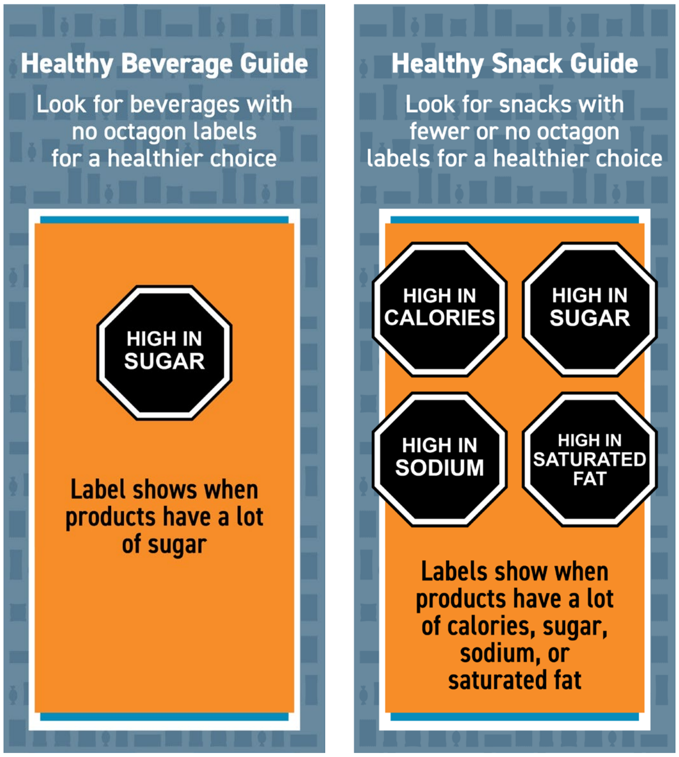


© 2023 Grummon AH et al. *JAMA Netw Open.*

**eTable 1. Characteristics of products offered in the trial[a]**

| Product | Product size (mL for beverages, g for snacks) | Calories (kcal) | Sugar (g) | Sat. fat (g) | Sodium (mg) | Green, yellow, or red label | Physical activity minutes equivalent | Calorie warning | Sugar warning | Sat. fat warning | Sodium warning |
|---|---|---|---|---|---|---|---|---|---|---|---|
| **Beverages** | | | | | | | | | | | |
| Soda 1 | 591.4 | 240 | 65 | 0 | 75 | Red | 108 | No | Yes | No | No |
| Soda 2 | 591.4 | 250 | 69 | 0 | 55 | Red | 112 | No | Yes | No | No |
| Soda 3 | 591.4 | 290 | 77 | 0 | 105 | Red | 130 | No | Yes | No | No |
| Sports drink | 591.4 | 140 | 34 | 0 | 270 | Red | 63 | No | Yes | No | No |
| Fruit drink | 591.4 | 260 | 67 | 0 | 80 | Red | 117 | No | Yes | No | No |
| Iced tea | 591.4 | 110 | 29 | 0 | 190 | Red | 49 | No | No | No | No |
| Diet soda 1 | 591.4 | 0 | 0 | 0 | 70 | Yellow | 0 | No | No | No | No |
| Diet soda 2 | 591.4 | 0 | 0 | 0 | 60 | Yellow | 0 | No | No | No | No |
| Diet soda 3 | 591.4 | 10 | 1 | 0 | 85 | Yellow | 4 | No | No | No | No |
| Diet sports drink | 591.4 | 50 | 12 | 0 | 270 | Yellow | 22 | No | No | No | No |
| 100% juice | 449.5 | 220 | 45 | 0 | 30 | Yellow | 99 | No | Yes | No | No |
| Diet iced tea | 591.4 | 0 | 0 | 0 | 210 | Yellow | 0 | No | No | No | No |
| Plain water | 591.4 | 0 | 0 | 0 | 0 | Green | 0 | No | No | No | No |
| Sparkling water 1 | 499.7 | 0 | 0 | 0 | 0 | Green | 0 | No | No | No | No |
| Sparkling water 2 | 591.4 | 0 | 0 | 0 | 0 | Green | 0 | No | No | No | No |
| Sparkling water 3 | 499.7 | 0 | 0 | 0 | 0 | Green | 0 | No | No | No | No |
| **Snacks** | | | | | | | | | | | |
| Candy 1 | 60.8 | 250 | 45 | 2.5 | 10 | Red | 112 | Yes | Yes | No | No |
| Candy 2 | 47.3 | 230 | 31 | 6 | 35 | Red | 103 | Yes | Yes | Yes | No |

© 2023 Grummon AH et al. *JAMA Netw Open.*

| Product | Product size (mL for beverages, g for snacks) | Calories (kcal) | Sugar (g) | Sat. fat (g) | Sodium (mg) | Green, yellow, or red label | Physical activity minutes equivalent | Calorie warning | Sugar warning | Sat. fat warning | Sodium warning |
|---|---|---|---|---|---|---|---|---|---|---|---|
| Candy 3 | 52.1 | 250 | 28 | 5 | 125 | Red | 112 | Yes | Yes | Yes | No |
| Chips 1 | 28.0 | 160 | 1 | 1.5 | 170 | Red | 72 | Yes | No | Yes | Yes |
| Cookies 1 | 84.0 | 400 | 34 | 5 | 330 | Red | 179 | Yes | Yes | Yes | No |
| Crackers | 28.0 | 150 | 4 | 3 | 135 | Yellow | 67 | Yes | No | Yes | No |
| Chips 2 | 28.0 | 140 | 0 | 1 | 210 | Yellow | 63 | Yes | No | No | Yes |
| Chips 3 | 28.0 | 140 | 2 | 1 | 200 | Yellow | 63 | Yes | No | No | Yes |
| Popcorn | 18.2 | 100 | 0 | 1 | 50 | Yellow | 45 | Yes | No | No | No |
| Granola bar 1 | 41.7 | 190 | 11 | 1 | 140 | Yellow | 85 | Yes | Yes | No | No |
| Granola bar 2 | 39.2 | 140 | 7 | 2 | 95 | Yellow | 63 | Yes | Yes | Yes | No |
| Trail mix | 56.0 | 270 | 14 | 3 | 5 | Yellow | 121 | Yes | Yes | No | No |
| Chips 4 | 24.5 | 120 | 1 | 1 | 210 | Green | 54 | Yes | No | No | Yes |
| Chips 5 | 31.5 | 135 | 2 | 0 | 152 | Green | 61 | Yes | No | No | No |
| Pretzels | 44.2 | 180 | 1 | 0 | 180 | Green | 81 | Yes | No | No | No |
| Cookies 2 | 22.7 | 100 | 8 | 0 | 150 | Green | 45 | Yes | Yes | No | Yes |

[a]Product brand names and flavors replaced with generic information for publication; participants saw branded products. Criteria for receiving red, yellow, or green labels and calorie, sugar, saturated fat, and sodium warning labels are described above. Note that a 20-ounce regular diet soda 3 contains 10 calories (as shown in the table) but the image of this diet soda used in the trial mistakenly displayed a “0 calorie” label in all arms.

© 2023 Grummon AH et al. *JAMA Netw Open.*

## eTable 2. Secondary psychological outcomes measured in the randomized trial[a]

| Outcome (range of response options) | Sample survey item |
|---|---|
| **Label reactions** | |
| Noticed label (0/1) | When you were selecting a beverage and a snack, did you notice any other nutrition labels [besides calorie labels] on or next to the products? |
| Attention to label (1–5) | How much does this label grab your attention? |
| Thinking about health effects (1–5) | How much does this label make you think about the health effects of foods and beverages? |
| Negative emotions (1–5) | How much does this label make you feel worried? |
| Discouragement from buying unhealthy foods (1–5) | How much does this label discourage you from wanting to buy unhealthy foods and beverages? |
| Encouragement to buy healthy foods (1–5) | How much does this label encourage you to want to buy healthy foods and beverages? |
| Label influenced beverage choice (0/1) | Did the label influence which beverage you selected? |
| Label influenced snack choice (0/1) | Did the label influence which snack you selected? |
| Message reactance (1–5) | This label is trying to manipulate me. |
| Healthfulness perceptions | |
| Healthfulness of healthy beverage (1–5) | How unhealthy or healthy do you think this beverage is? [Show image of healthy beverage] |
| Healthfulness of moderately healthy beverage (1–5) | How unhealthy or healthy do you think this beverage is? [Show image of moderately healthy beverage] |
| Healthfulness of unhealthy beverage (1–5) | How unhealthy or healthy do you think this beverage is? [Show image of unhealthy beverage] |
| Healthfulness of healthy snack (1–5) | How unhealthy or healthy do you think this product is? [Show image of healthy snack] |
| Healthfulness of moderately healthy snack (1–5) | How unhealthy or healthy do you think this product is? [Show image of moderately healthy snack] |
| Healthfulness of unhealthy snack (1–5) | How unhealthy or healthy do you think this product is? [Show image of unhealthy snack] |
| Stigma | |
| Perceived personal stigma (1–5) | How personally stigmatized does this label make you feel? |
| Perceived obesity stigma (1–5) | This label stigmatizes people with obesity. |
| Disgust toward people with obesity (1–5) | How disgusted do people with obesity make you feel? |
| Positive label perceptions | |
| Learned something new from the label (1–5) | To what extent did you learn something new from this label? |
| Trust information in the label (1–5) | How much do you trust or distrust the information in this label? |
| Perceptions of control over healthy eating decisions (1–3, recoded in analysis) | Overall, does this label make you feel less in control, neither less nor more in control, or more in control of making healthy eating decisions? |
| Used label to help choose beverage (1–5) | How much did you use this label to help you decide which beverage to select? |
| Used label to help choose snack (1–5) | How much did you use this label to help you decide which snack to select? |

[a]Full survey measures are shown in **eTable 3.**

## eTable 3. Survey measures used in the trial

| Construct | Item | Response scale | Reference |
|---|---|---|---|
| | **Introduction** | | |
| Consent | On this page, we describe the consent process.<br><br>1. First, it's important to know your participation is completely voluntary. You can stop participating at any time.<br><br>2. Second, your information will be de-identified. De-identified means that all identifiers have been removed. The information could be stored and shared for future research in this de-identified fashion. It would not be possible for future researchers to identify you as we would not share any identifiable information about you with future researchers. This can be done without again seeking your consent in the future, as permitted by law. The future use of your information only applies to the information collected on this study.<br><br>3. Third, we are conducting consumer market research about shopping experiences and behaviors. We are asking for your permission to tell you exactly what we are studying once the research has ended. At that point, you will have the chance to tell us whether we can use your data or whether you prefer we destroy it and not include it in our study.<br><br>4. Fourth, we wanted to let you know that 100 respondents will be selected at random for a "bonus" in which you will have one of your product selections delivered to your home address.<br><br>Do you consent to be part of the research study? | 1=Yes<br>0=No | |
| Captcha | Please complete the captcha.<br><br>[display Captcha button] | | |
| | **Initial Instructions for Vending Machine Selection Tasks** | | |

© 2023 Grummon AH et al. *JAMA Netw Open.*

| Construct | Item | Response scale | Reference |
|---|---|---|---|
| Prompt | In this survey, you will see beverages and snacks you might see in a vending machine. We will ask you to select the beverage and the snack you would most like to purchase. We will randomly select 100 respondents to receive a "bonus" for completing this part of the survey. If you are selected for the bonus, you will have one of your product selections delivered to you.<br><br>You should choose the beverage and snack you most want to purchase because your choices could be delivered to you.<br><br>[randomly assign participants to a front-of-package labeling condition: control; green labels, traffic light labels, physical activity labels, or nutrient warning labels]<br><br>[randomize order of beverage vs. snack choice task] | | |
| | **Beverage Selection Task** | | |
| Prompt | The next section is about beverage choices. You will see beverages you might see in a vending machine. We will ask you to select the beverage you would most like to purchase.<br><br>If you see a beverage brand you want but don't see the flavor you want, please still select that beverage regardless of the flavor available. | | |
| Prompt | [Display only for Green condition]<br><br>[Insert green labels beverage poster] | | |
| Prompt | [Display only for traffic light labels condition]<br><br>[Insert traffic light labels beverage poster] | | |
| Prompt | [Display only for physical activity labels condition]<br><br>[Insert physical activity labels beverage poster] | | |
| Prompt | [Display only for nutrient warning labels condition]<br><br>[Insert nutrient warning labels beverage poster] | | (Roberto et al., 2016) |
| Beverage choice task | Imagine you are looking at a vending machine because you want to purchase a beverage. Take a few minutes to look at the beverages below. Make sure to scroll to see all of the options. Then, click on the item you would most like to purchase. | [display beverage images from participant's condition, shown in a random arrangement] | Adapted from (Roberto et al., 2016) |
| | **Snack Selection Task** | | |
| Prompt | The next section is about snack choices. You will see snacks you might see in a vending machine. We will ask you to select the snack you would most like to purchase.<br><br>If you see a snack brand you want but don't see the flavor you want, please still select that snack regardless of the flavor available. | | |

© 2023 Grummon AH et al. *JAMA Netw Open.*

| Construct | Item | Response scale | Reference |
|---|---|---|---|
| Prompt | [Display only for Green condition]<br><br>[Insert green labels snack poster] | | |
| Prompt | [Display only for traffic light labels condition]<br><br>[Insert traffic light labels snack poster] | | |
| Prompt | [Display only for physical activity labels condition]<br><br>[Insert physical activity labels snack poster] | | |
| Prompt | [Display only for nutrient warning labels condition]<br><br>[Insert nutrient warning labels snack poster] | | |
| Snack choice task | Imagine you are looking at a vending machine because you want to purchase a snack. Take a few minutes to look at the products below. Make sure to scroll to see all of the options. Then, click on the item you would most like to purchase. | [display snack images from participant's condition, shown in a random arrangement] | Adapted from (Roberto et al., 2016) |
| | **Healthfulness Perceptions – Beverages** | | |
| Prompt | The next questions are about different beverages.<br><br>[randomize order of beverages] | | |
| Perceived healthfulness-unhealthy item | How unhealthy or healthy do you think this beverage is?<br><br>[insert image of [brand name redacted for publication] with label from participant's condition] | 1=Very unhealthy<br>2=Somewhat unhealthy<br>3=Neither unhealthy nor healthy<br>4=Somewhat healthy<br>5=Very healthy | Adapted from (Bollard et al., 2016) |
| Perceived healthfulness-somewhat unhealthy item | How unhealthy or healthy do you think this beverage is?<br><br>[insert image of [brand name redacted for publication] with label from participant's condition] | 1=Very unhealthy<br>2=Somewhat unhealthy<br>3=Neither unhealthy nor healthy<br>4=Somewhat healthy<br>5=Very healthy | Adapted from (Bollard et al., 2016) |
| Perceived healthfulness-healthy item | How unhealthy or healthy do you think this beverage is?<br><br>[insert image of [brand name redacted for publication] with label from participant's condition] | 1=Very unhealthy<br>2=Somewhat unhealthy<br>3=Neither unhealthy nor healthy<br>4=Somewhat healthy<br>5=Very healthy | Adapted from (Bollard et al., 2016) |
| | **Healthfulness Perceptions – Snacks** | | |
| Prompt | The next questions are about different snacks.<br><br>[randomize order of snacks] | | |
| Perceived healthfulness-unhealthy item | How unhealthy or healthy do you think this product is?<br><br>[insert image of [brand name redacted for publication] with label from participant's condition] | 1=Very unhealthy<br>2=Somewhat unhealthy<br>3=Neither unhealthy nor healthy<br>4=Somewhat healthy<br>5=Very healthy | Adapted from (Bollard et al., 2016) |
| Perceived healthfulness-somewhat unhealthy item | How unhealthy or healthy do you think this product is?<br><br>[insert image of [brand name redacted for publication] with label from participant's condition] | 1=Very unhealthy<br>2=Somewhat unhealthy<br>3=Neither unhealthy nor healthy<br>4=Somewhat healthy<br>5=Very healthy | Adapted from (Bollard et al., 2016) |

© 2023 Grummon AH et al. *JAMA Netw Open.*

| Construct | Item | Response scale | Reference |
|---|---|---|---|
| Perceived healthfulness-healthy item | How unhealthy or healthy do you think this product is?<br><br>[insert image of [brand name redacted for publication] with label from participant's condition] | 1=Very unhealthy<br>2=Somewhat unhealthy<br>3=Neither unhealthy nor healthy<br>4=Somewhat healthy<br>5=Very healthy | Adapted from (Bollard et al., 2016) |
| | **Label Reactions and Perceptions**<br>[For participants in the traffic light labels or nutrient warnings labels arms, label reaction and perception items replaced "this label" with "these labels" and used plural conjugation as needed] | | |
| Noticing calorie labels | When you were selecting a beverage and a snack, did you notice any calorie labels on or next to the products? | 1=Yes<br>0=No<br>2=Not sure | |
| Noticing other labels | When you were selecting a beverage and a snack, did you notice any other nutrition labels on or next to the products? | 1=Yes<br>0=No<br>2=Not sure | |
| Prompt & image | Below is a picture of a label that was on some of the products you saw when you selected a beverage and snack. The next questions are about this type of label.<br><br>[display participants' randomly assigned label or labels] | | |
| Label influenced beverage choice | Did the label influence which beverage you selected? | 1=Yes<br>0=No<br>2=Did not see the label | |
| Used label to help choose beverage | How much did you use this label to help you decide which beverage to select?<br><br>[display participants' randomly assigned label or labels] | 1=Not at all<br>2=Very little<br>3=Somewhat<br>4=Quite a bit<br>5=A great deal | |
| Label influenced snack choice | Did the label influence which snack you selected?<br><br>[display participants' randomly assigned label or labels] | 1=Yes<br>0=No<br>2=Did not see the label | |
| Used label to help choose snack | How much did you use this label to help you decide which snack to select?<br><br>[display participants' randomly assigned label or labels] | 1=Not at all<br>2=Very little<br>3=Somewhat<br>4=Quite a bit<br>5=A great deal | |
| Attention to label | How much does this label grab your attention?<br><br>[display participants' randomly assigned label or labels] | 1=Not at all<br>2=Very little<br>3=Somewhat<br>4=Quite a bit<br>5=A great deal | (Nonnemaker et al., 2010) |
| Thinking about health effects | How much does this label make you think about the health effects of foods and beverages?<br><br>[display participants' randomly assigned label or labels] | 1=Not at all<br>2=Very little<br>3=Somewhat<br>4=Quite a bit<br>5=A great deal | Adapted from (Fathelrahman et al., 2010) and (Hammond et al., 2003) |

© 2023 Grummon AH et al. *JAMA Netw Open.*

| Construct | Item | Response scale | Reference |
|---|---|---|---|
| Perceived message effectiveness – discouragement from buying unhealthy foods | How much does this label discourage you from wanting to buy unhealthy foods and beverages?<br><br>[display participants' randomly assigned label or labels] | 1=Not at all<br>2=Very little<br>3=Somewhat<br>4=Quite a bit<br>5=A great deal | Adapted from (Baig et al., 2019) |
| Perceived message effectiveness – encouragement to buy healthy foods | How much does this label encourage you to want to buy healthy foods and beverages?<br><br>[display participants' randomly assigned label or labels] | 1=Not at all<br>2=Very little<br>3=Somewhat<br>4=Quite a bit<br>5=A great deal | Newly developed based on (Baig et al., 2019) |
| Negative emotions – stem | [display participants' randomly assigned label or labels; format as matrix and randomize order in which emotions are displayed]<br><br>How much does this label make you feel… | 1=Not at all<br>2=Very little<br>3=Somewhat<br>4=Quite a bit<br>5=A great deal | (Brewer et al., 2019) |
| Negative emotions –worry | worried? | | |
| Negative emotions – fear | scared? | | |
| Negative emotions – guilt | guilty? | | |
| Negative emotions – shame | ashamed? | | |
| Negative emotions – sadness | sad? | | |
| Learn something new | To what extent did you learn something new from this label?<br><br>[display participants' randomly assigned label or labels] | 1=Not at all<br>2=Very little<br>3=Somewhat<br>4=Quite a bit<br>5=A great deal | Adapted from (RTI International, 2018) |
| Trust information in the label | How much do you trust or distrust the information in this label?<br><br>[display participants' randomly assigned label or labels] | 1=Completely distrust<br>2=Somewhat distrust<br>3=Neither trust nor distrust<br>4=Somewhat trust<br>5=Completely trust | |
| Perceptions of control over healthy eating decisions | Overall, does this label make you feel…<br><br>[display participants' randomly assigned label or labels] | 1=Less in control of making healthy eating decisions<br>2=Neither less nor more in control of making healthy eating decisions<br>3=More in control of making healthy eating decisions | (Acton & Hammond, 2018) |

| Construct | Item | Response scale | Reference |
|---|---|---|---|
| Reactance – manipulation | This label is trying to manipulate me.<br><br>[display participants' randomly assigned label or labels] | 1=Not at all<br>2=Very little<br>3=Somewhat<br>4=Quite a bit<br>5=A great deal | (Hall et al., 2017) |
| Reactance – annoyance | This label annoys me.<br><br>[display participants' randomly assigned label or labels] | 1=Not at all<br>2=Very little<br>3=Somewhat<br>4=Quite a bit<br>5=A great deal | (Hall et al., 2017) |
| Perceived personal stigma | How personally stigmatized does this label make you feel?<br><br>[display participants' randomly assigned label or labels] | 1=Not at all<br>2=Very little<br>3=Somewhat<br>4=Quite a bit<br>5=A great deal | (Hayward & Vartanian, 2019) |
| Perceived obesity stigma | This label stigmatizes people with obesity.<br><br>[display participants' randomly assigned label or labels] | 1=Not at all<br>2=Very little<br>3=Somewhat<br>4=Quite a bit<br>5=A great deal | Adapted from (Puhl et al., 2013) |
| Perceived obesity stigma | This label increases blame toward people for being overweight.<br><br>[display participants' randomly assigned label or labels] | 1=Not at all<br>2=Very little<br>3=Somewhat<br>4=Quite a bit<br>5=A great deal | Adapted from (Puhl et al., 2013) |
| Disgust toward people with obesity | How disgusted do people with obesity make you feel?<br><br>[display participants' randomly assigned label or labels] | 1=Not at all<br>2=Very little<br>3=Somewhat<br>4=Quite a bit<br>5=A great deal | Adapted from (Hayward & Vartanian, 2019) |
| | **Demographics** | | |
| Prompt | We are asking the questions in the next section to better understand who completed our survey. | | |
| Age | How old are you? | [free response, #, restricted to 18-115] | |
| Gender | How do you identify? | 1=Woman<br>2=Man<br>3=Non-binary<br>4=Prefer to self-describe:_____ | |
| Hispanic/Latino ethnicity | Are you of Hispanic, Latino, or Spanish origin? | 1=Yes<br>0=No | 2010 Census |
| Race | What is your race? (Check all that apply). | [Check all that apply]<br>1=American Indian or Alaska Native<br>2=Asian<br>3=Black or African American<br>4=Native Hawaiian or Other Pacific Islander<br>5=White<br>6=Another race: | 2010 Census |

© 2023 Grummon AH et al. *JAMA Netw Open.*

| Construct | Item | Response scale | Reference |
|---|---|---|---|
| Education | What is the highest degree or level of school you have completed? | 1=Less than high school<br>2=High school graduate (or GED)<br>3=Some college or technical school<br>4=Associate's degree<br>5=Bachelor's degree<br>6=Graduate or professional degree | |
| Diet quality | In general, how healthy is your overall diet? Would you say… | 1=Poor<br>2=Fair<br>3=Good<br>4=Very good<br>5=Excellent | (Adjoian et al., 2016) |
| Numeracy | The following question asks about mental math. Please answer it to the best of your ability.<br><br>Out of 1,000 people in a small town 500 are members of a choir. Out of these 500 members in the choir 100 are men. Out of the 500 inhabitants that are not in the choir 300 are men. What is the probability that a randomly drawn man is a member of the choir? | Please indicate the probability as a percent (e.g., if you would like to enter 100%, enter the number "100" in the box): [free response box, restrict to 0-100, integers only (no decimals)] | |
| Health literacy | How often do you need to have someone help you when you read instructions, pamphlets, or other written materials from your doctor or pharmacy? | 1=Never<br>2=Rarely<br>3=Sometimes<br>4=Most of the time<br>5=Always | |
| Nutrition Label Use | How often do you use the Nutrition Facts label when deciding to buy a food product? | 1=Never<br>2=Rarely<br>3=Sometimes<br>4=Most of the time<br>5=Always | Adapted from (Centers for Disease Control and Prevention National Center for Health Statistics, 2016) |
| Frequency of buying beverages from vending machines | In a typical week, how often do you buy <u>beverages</u> from vending machines? | 1=Never or less than 1 time per week<br>2=1 to 2 times per week<br>3 = 3 to 4 times per week<br>4 = 5 or more times per week | |
| Frequency of buying snacks from vending machines | In a typical week, how often do you buy <u>snacks</u> from vending machines? | 1=Never or less than 1 time per week<br>2=1 to 2 times per week<br>3 = 3 to 4 times per week<br>4 = 5 or more times per week | |

© 2023 Grummon AH et al. *JAMA Netw Open.*

| Construct | Item | Response scale | Reference |
|---|---|---|---|
| Party identification | Do you consider yourself a Democrat, a Republican, an Independent, or something else? | 1=Strong Democrat<br>2=Moderate Democrat<br>3=Lean Democrat<br>4=Independent<br>5=Lean Republican<br>6=Moderate Republican<br>7=Strong Republican<br>8=Something else:____ | |
| Household size | How many people are in your household, including you? | [# of people [restricted to 1-20, whole numbers] | |
| Children | How many children (ages 0-18) currently live in your household? | ____ [#, restricted to 0-15, whole numbers] | |
| Height, feet | How tall are you? Please enter your height in **feet** and **inches.** | [Drop down menu]<br>3 = 3 feet<br>4 = 4 feet<br>5 = 5 feet<br>6 = 6 feet<br>7 = 7 feet<br>8 = 8 feet | |
| Height, inches | [Show on same page as above] | [Drop down menu]<br>0=0 inches<br>1 = 1 inch<br>2 = 2 inches<br>3 = 3 inches<br>4 = 4 inches<br>5 = 5 inches<br>6 = 6 inches<br>7 = 7 inches<br>8 = 8 inches<br>9 = 9 inches<br>10 = 10 inches<br>11 = 11 inches | |
| Weight, lbs | How much do you weigh? Enter your weight in **pounds**. | [Numerical free response. Allow entries ≥40lbs and ≤900lbs).]<br>____ pounds | |
| Income, dollar categories | Which of the following categories best describes your total household income before taxes in the last 12 months? | 1=Less than $10,000<br>2=$10,000 to $14,999<br>3=$15,000 to $24,999<br>4=$25,000 to $34,999<br>5=$35,000 to $49,999<br>6=$50,000 to $74,999<br>7=$75,000 to $99,999<br>8=$100,000 to $149,999<br>9=$150,000 to $199,999<br>10=$200,000 or more | |

© 2023 Grummon AH et al. *JAMA Netw Open.*

| Construct | Item | Response scale | Reference |
|---|---|---|---|
| Income, poverty line categories | [Ask only if household size is 1]<br>Which of the following categories best describes your total household income before taxes in the last 12 months? | 1=Less than $19,140<br>2=Between $19,140 and $25,519<br>3=Between $25,520 and $31,899<br>4=Between $31,900 and $38,279<br>5=$38,280 or more | |
| Income, poverty line categories | [Ask only if household size is 2]<br>Which of the following categories best describes your total household income before taxes in the last 12 months? | 1=Less than $25,860<br>2=Between $25,860 and $34,479<br>3=Between $34,480 and $43,099<br>4=Between $43,100 and $51,719<br>5=$51,720 or more | |
| Income, poverty line categories | [Ask only if household size is 3]<br>Which of the following categories best describes your total household income before taxes in the last 12 months? | 1=Less than $32,580<br>2=Between $32,580 and $43,439<br>3=Between $43,440 and $54,299<br>4=Between $54,300 and $65,159<br>5=$65,160 or more | |
| Income, poverty line categories | [Ask only if household size is 4]<br>Which of the following categories best describes your total household income before taxes in the last 12 months? | 1=Less than $39,300<br>2=Between $39,300 and $52,399<br>3=Between $52,400 and $65,499<br>4=Between $65,500 and $78,599<br>5=$78,600 or more | |
| Income, poverty line categories | [Ask only if household size is 5]<br>Which of the following categories best describes your total household income before taxes in the last 12 months? | 1=Less than $46,020<br>2=Between $46,020 and $61,359<br>3=Between $61,360 and $76,699<br>4=Between $76,700 and $92,039<br>5=$92,040 or more | |
| Income, poverty line categories | [Ask only if household size is 6]<br>Which of the following categories best describes your total household income before taxes in the last 12 months? | 1=Less than $52,740<br>2=Between $52,740 and $70,319<br>3=Between $70,320 and $87,899<br>4=Between $87,900 and $105,479<br>5=$105,480 or more | |
| Income, poverty line categories | [Ask only if household size is 7]<br>Which of the following categories best describes your total household income before taxes in the last 12 months? | 1=Less than $59,460<br>2=Between $59,460 and $79,279<br>3=Between $79,280 and $99,099<br>4=Between $99,100 and $118,919<br>5=$118,920 or more | |

© 2023 Grummon AH et al. *JAMA Netw Open.*

| Construct | Item | Response scale | Reference |
|---|---|---|---|
| Income, poverty line categories | [Ask only if household size is 8]<br>Which of the following categories best describes your total household income before taxes in the last 12 months? | 1=Less than $66,180<br>2=Between $66,180 and $88,239<br>3=Between $88,240 and $110,299<br>4=Between $110,300 and $132,359<br>5=$132,360 or more | |
| Income, poverty line categories | [Ask only if household size is 9]<br>Which of the following categories best describes your total household income before taxes in the last 12 months? | 1=Less than $72,900<br>2=Between $72,900 and $97,199<br>3=Between $97,200 and $121,499<br>4=Between $121,500 and $145,799<br>5=$145,800 or more | |
| Income, poverty line categories | [Ask only if household size is 10]<br>Which of the following categories best describes your total household income before taxes in the last 12 months? | 1=Less than $79,620<br>2=Between $79,620 and $106,159<br>3=Between $106,160 and $132,699<br>4=Between $132,700 and $159,239<br>5=$159,240 or more | |
| Income, poverty line categories | [Ask only if household size is 11]<br>Which of the following categories best describes your total household income before taxes in the last 12 months? | 1=Less than $86,340<br>2=Between $86,340 and $115,119<br>3=Between $115,120 and $143,899<br>4=Between $143,900 and $172,679<br>5=$172,680 or more | |
| Income, poverty line categories | [Ask only if household size is 12]<br>Which of the following categories best describes your total household income before taxes in the last 12 months? | 1=Less than $93,060<br>2=Between $93,060 and $124,079<br>3=Between $124,080 and $155,099<br>4=Between $155,100 and $186,119<br>5=$186,120 or more | |
| Income, poverty line categories | [Ask only if household size is 13]<br>Which of the following categories best describes your total household income before taxes in the last 12 months? | 1=Less than $99,780<br>2=Between $99,780 and $133,039<br>3=Between $133,040 and $166,299<br>4=Between $166,300 and $199,559<br>5=$199,560 or more | |
| Income, poverty line categories | [Ask only if household size is 14]<br>Which of the following categories best describes your total household income before taxes in the last 12 months? | 1=Less than $106,500<br>2=Between $106,500 and $141,999<br>3=Between $142,000 and $177,499<br>4=Between $177,500 and $212,999<br>5=$213,000 or more | |

© 2023 Grummon AH et al. *JAMA Netw Open.*

| Construct | Item | Response scale | Reference |
|---|---|---|---|
| Income, poverty line categories | [Ask only if household size is 15]<br>Which of the following categories best describes your total household income before taxes in the last 12 months? | 1=Less than $113,220<br>2=Between $113,220 and $150,959<br>3=Between $150,960 and $188,699<br>4=Between $188,700 and $226,439<br>5=$226,440 or more | |
| Income, poverty line categories | [Ask only if household size is 16]<br>Which of the following categories best describes your total household income before taxes in the last 12 months? | 1=Less than $119,940<br>2=Between $119,940 and $159,919<br>3=Between $159,920 and $199,899<br>4=Between $199,900 and $239,879<br>5=$239,880 or more | |
| Income, poverty line categories | [Ask only if household size is 17]<br>Which of the following categories best describes your total household income before taxes in the last 12 months? | 1=Less than $126,660<br>2=Between $126,660 and $168,879<br>3=Between $168,880 and $211,099<br>4=Between $211,100 and $253,319<br>5=$253,320 or more | |
| Income, poverty line categories | [Ask only if household size is 18]<br>Which of the following categories best describes your total household income before taxes in the last 12 months? | 1=Less than $133,380<br>2=Between $133,380 and $177,839<br>3=Between $177,840 and $222,299<br>4=Between $222,300 and $266,759<br>5=$266,760 or more | |
| Income, poverty line categories | [Ask only if household size is 19]<br>Which of the following categories best describes your total household income before taxes in the last 12 months? | 1=Less than $140,100<br>2=Between $140,100 and $186,799<br>3=Between $186,800 and $233,499<br>4=Between $233,500 and $280,199<br>5=$280,200 or more | |
| Income, poverty line categories | [Ask only if household size is 20]<br>Which of the following categories best describes your total household income before taxes in the last 12 months? | 1=Less than $146,820<br>2=Between $146,820 and $195,759<br>3=Between $195,760 and $244,699<br>4=Between $244,700 and $293,639<br>5=$293,640 or more | |
| | **Closure** | | |
| Final comments | Is there anything else you want to tell us? | [Open ended] | |

© 2023 Grummon AH et al. *JAMA Netw Open.*

| Construct | Item | Response scale | Reference |
|---|---|---|---|
| Debrief | Thank you for taking part in this research study. There was some information about the study that we did not share with you at the beginning of your participation, so that you would react honestly. We would now like to fully inform you about the nature of this research and explain a bit more about the survey you just completed.<br><br>In the survey we indicated that 100 participants would receive one of the foods/beverage products they selected from the vending machines in the survey shipped directly to their mailing address in addition to the amount agreed upon when they entered this survey. In actuality, all participants selected for this bonus will receive a $2.50 gift card (slightly more than the value of the most expensive item we showed you in the survey) in addition to the amount agreed upon when you entered this survey.<br><br>We stated that you might receive one of the foods/beverage products you selected because we wanted you to behave as you normally would when choosing foods and to choose options you would actually want to purchase. This study was conducted by researchers at the University of Pennsylvania and Harvard University who are interested in the effects of nutrition and warning labels on food and beverage purchases. We will use this study to determine how effective different types of messages and front-of-package labels are at changing which products people choose.<br><br>Please feel free to contact the investigator, Anna Grummon, with any questions or if you have any concerns about your participation. She can be reached via email at agrummon@hsph.harvard.edu. If you would like a copy of this debriefing form, please save this page to your computer.<br><br>Please choose one of the options below. | 1=I agree to have my data included in this study.<br>0=I no longer wish to participate and/or I would like my data removed from the study. (Your data will be removed from the study entirely, except for the consent and debriefing form). | |
| Debrief | Please confirm that you would like your data removed from the study. | 0=Confirm, remove my data from the study<br>1=Keep my data in the study | |
| Closure | You have now finished this survey. Thank you for your participation! | | |

© 2023 Grummon AH et al. *JAMA Netw Open.*

**eTable 4. Comparison of characteristics of the study sample (n=7,945 U.S. adults) to national estimates[a]**

| | Study sample % | National estimate % |
|---|---|---|
| Age | | |
| 18-29 years | 20% | 20% |
| 30-44 years | 25% | 26% |
| 45-59 years | 25% | 24% |
| 60 years or older | 30% | 30% |
| Gender | | |
| Female | 51% | 51% |
| Male | 48% | 49% |
| Non-binary or another gender | 1% | -- |
| Latino(a) or Hispanic | 16% | 17% |
| Race | | |
| American Indian or Alaska Native | 1% | 1% |
| Asian or Pacific Islander | 3% | 6% |
| Black or African American | 12% | 12% |
| White | 76% | 64% |
| Other or Multiracial | 7% | 18% |
| Education | | |
| High school diploma or less | 27% | 38% |
| Some college | 23% | 21% |
| College graduate or associate degree | 39% | 29% |
| Graduate degree | 11% | 12% |
| Self-rated diet quality | | |
| Poor | 6% | 6% |
| Fair | 30% | 24% |
| Good | 40% | 41% |
| Very good | 18% | 22% |
| Excellent | 6% | 8% |
| Household income, annual | | |
| $0 to $24,999 | 26% | 17% |
| $25,000 to $49,999 | 29% | 19% |
| $50,000 to $74,999 | 19% | 16% |
| $75,000 or more | 26% | 48% |

[a]National estimates for age, gender/sex, ethnicity, race, and education are survey-weighted estimates among adults (ages 18-years and older) in the 2021 American Community Survey (ACS) 1-year Public Use Microdata Sample (PUMS) (United States Census Bureau, 2022). National estimate of % of people identifying as non-binary or another gender is listed as "NA" because the ACS only includes information on identification as male or female. National estimates for self-rated dietary quality are from the National Health and Nutrition Examination Surveys cycles 2015–2018, as reported by Thomson and colleagues (2022). National estimates for income are from the Current Population Survey, 2021, the most recent data currently available (Semega & Kollar, 2022).

**eTable 5. Calories, nutrients, and type of items selected in beverage and snack selection tasks by trial arm, *n*=7,945[a]**

| | Control *n*=1,593 | Green labels *n*=1,587 | Single traffic light labels *n*=1,588 | Physical activity labels *n*=1,590 | Nutrient warning labels *n*=1,587 |
|---|---|---|---|---|---|
| | **Mean (SD)** | **Mean (SD)** | **Mean (SD)** | **Mean (SD)** | **Mean (SD)** |
| **Beverage selection** | | | | | |
| Calories selected (kcal) | 125.8 (116.8) | 91.7 (113.6) | 94.3 (115.3) | 86.8 (111.7) | 97.6 (113.9) |
| Sugar selected (g) | 32.9 (31.2) | 23.8 (30.0) | 24.4 (30.4) | 22.4 (29.5) | 25.4 (30.3) |
| Sodium selected (mg) | 93.8 (82.4) | 81.0 (85.1) | 70.5 (81.6) | 87.9 (88.3) | 93.0 (87.6) |
| Selected a healthy item, n (%) | 311 (20) | 526 (33) | 601 (38) | 473 (30) | 412 (26) |
| Selected a moderately healthy item, n (%) | 459 (29) | 471 (30) | 420 (26) | 564 (35) | 549 (35) |
| Selected an unhealthy item, n (%) | 822 (52) | 590 (37) | 567 (36) | 552 (35) | 626 (39) |
| **Snack selection** | | | | | |
| Calories selected (kcal) | 182.8 (69.2) | 170.1 (61.6) | 169.1 (62.9) | 164.3 (68.4) | 168.6 (67.2) |
| Sugar selected (g) | 11.5 (14.4) | 9.2 (13.2) | 8.4 (12.7) | 9.2 (13.1) | 8.7 (13.5) |
| Sodium selected (mg) | 149.6 (74.1) | 152.5 (66.8) | 153.2 (68.0) | 142.0 (72.8) | 144.0 (73.9) |
| Saturated fat selected (g) | 2.2 (1.9) | 1.8 (1.8) | 1.6 (1.7) | 1.7 (1.8) | 1.6 (1.8) |
| Selected a healthy item, n (%) | 268 (17) | 480 (30) | 494 (31) | 384 (24) | 427 (27) |
| Selected a moderately healthy item, n (%) | 677 (42) | 619 (39) | 673 (42) | 733 (46) | 706 (44) |
| Selected an unhealthy item, n (%) | 648 (41) | 488 (31) | 421 (27) | 473 (30) | 454 (29) |

[a]Table shows means (SD) or percentages (n) of selection outcomes by trial arm. We did not examine saturated fat for beverages because none of the beverages contained saturated fat. There were missing data for *n*=2 beverage selections (<0.1%).

© 2023 Grummon AH et al. *JAMA Netw Open.*

## eTable 6. Effects of interpretative front-of-package labeling systems on beverage and snack selections, n=7,945[a]

| | Green labels *n*=1,587 vs. control *n*=1,593 | | | Single traffic light labels *n*=1,588 vs. control *n*=1,593 | | | Physical activity labels *n*=1,590 vs. control *n*=1,593 | | | Nutrient warning labels *n*=1,587 vs. control *n*=1,593 | | |
|---|---|---|---|---|---|---|---|---|---|---|---|---|
| **Beverage selection** | **ADE** | **(95% CI)** | **Corr. *p*** | **ADE** | **(95% CI)** | **Corr. *p*** | **ADE** | **(95% CI)** | **Corr. *p*** | **ADE** | **(95% CI)** | **Corr. *p*** |
| Calories selected (kcal) | **-34.2[a,b]** | **(-42.2, -26.1)** | **<.001** | **-31.5[a,b]** | **(-39.5, -23.4)** | **<.001** | **-39.0[a]** | **(-47.0, -31.1)** | **<.001** | **-28.2[b]** | **(-36.2, -20.2)** | **<.001** |
| Sugar selected (g) | **-9.1[a,b]** | **(-11.3, -7.0)** | **<.001** | **-8.5[a,b]** | **(-10.7, -6.4)** | **<.001** | **-10.5[a]** | **(-12.6, -8.4)** | **<.001** | **-7.5[b]** | **(-9.6, -5.4)** | **<.001** |
| Sodium selected (mg) | **-12.8[a]** | **(-18.7, -6.9)** | **<.001** | **-23.3[b]** | **(-29.2, -17.4)** | **<.001** | -5.9[c] | (-11.8, 0.0) | .10 | -0.8[c] | (-6.7, 5.1) | .79 |
| Likelihood of selecting a healthy item | **13.6%[a]** | **(10.6%, 16.6%)** | **<.001** | **18.3%[b]** | **(15.2%, 21.4%)** | **<.001** | **10.2%[c]** | **(7.3%, 13.2%)** | **<.001** | **6.4%[d]** | **(3.5%, 9.3%)** | **<.001** |
| Likelihood of selecting a moderately healthy item | 0.8%[a] | (-2.3%, 4.0%) | .60 | -2.4%[a] | (-5.5%, 0.7%) | .27 | **6.7%[b]** | **(3.4%, 9.9%)** | **<.001** | **5.8%[b]** | **(2.5%, 9.0%)** | **.001** |
| Likelihood of selecting an unhealthy item | **-14.5%[a,b]** | **(-17.9%, -11.0%)** | **<.001** | **-15.9%[a,b]** | **(-19.3%, -12.5%)** | **<.001** | **-16.9%[a]** | **(-20.3%, -13.5%)** | **<.001** | **-12.2%[b]** | **(-15.6%, -8.8%)** | **<.001** |
| **Snack selection** | | | | | | | | | | | | |
| Calories selected (kcal) | **-12.7[a]** | **(-17.3, -8.2)** | **<0.001** | **-13.7[a]** | **(-18.2, -9.1)** | **<.001** | **-18.5[a]** | **(-23.1, -13.9)** | **<.001** | **-14.2[a]** | **(-18.8, -9.6)** | **<.001** |
| Sugar selected (g) | **-2.3[a]** | **(-3.2, -1.3)** | **<0.001** | **-3.1[a]** | **(-4.0, -2.1)** | **<.001** | **-2.3[a]** | **(-3.3, -1.4)** | **<.001** | **-2.8[a]** | **(-3.8, -1.8)** | **<.001** |
| Sodium selected (mg) | 2.9[a] | (-2.0, 7.9) | .25 | 3.7[a] | (-1.3, 8.6) | .29 | **-7.6[b]** | **(-12.5, -2.6)** | **.01** | -5.6[b] | (-10.6, -0.7) | .08 |
| Saturated fat selected (g) | **-0.4[a]** | **(-0.6, -0.3)** | **<.001** | **-0.6[a]** | **(-0.7, -0.5)** | **<.001** | **-0.5[a]** | **(-0.6, -0.4)** | **<.001** | **-0.6[a]** | **(-0.7, -0.5)** | **<.001** |
| Likelihood of selecting a healthy item | **13.4%[a,c]** | **(10.5%, 16.3%)** | **<.001** | **14.3%[a]** | **(11.4%, 17.2%)** | **<.001** | **7.3%[b]** | **(4.5%, 10.1%)** | **<.001** | **10.1%[b,c]** | **(7.2%, 12.9%)** | **<.001** |
| Likelihood of selecting a moderately healthy item | -3.5%[a] | (-6.9%, -0.1%) | .13 | -0.1%[a,b] | (-3.6%, 3.3%) | .95 | 3.6%[b] | (0.2%, 7.1%) | .16 | 2.0%[b] | (-1.5%, 5.4%) | .52 |
| Likelihood of selecting an unhealthy item | **-9.9%[a]** | **(-13.2%, -6.6%)** | **<.001** | **-14.2%[b]** | **(-17.4%, -10.9%)** | **<.001** | **-10.9%[a,b]** | **(-14.2%, -7.6%)** | **<.001** | **-12.1%[a,b]** | **(-15.4%, -8.8%)** | **<.001** |

[a]ADE, average differential effect, Corr. *p*, corrected *p*-value. **Bold** indicates a statistically significant impact of the interpretative front-of-package labeling arm (green, single traffic light, physical activity, or nutrient warning label) compared to the control arm, Holm-Bonferroni-corrected $p<.05$ (4 tests per outcome, each interpretative label vs. control). ADEs sharing a superscript did not differ from one another, Holm-Bonferroni-corrected $p \geq 0.05$ (6 tests to compare the effects of the 4 interpretive labels to one another). We did not examine saturated fat for beverages because none of the beverages contained saturated fat. There were missing data for *n*=2 beverage selections (<0.1%).

© 2023 Grummon AH et al. *JAMA Netw Open.*

**eTable 7. Effects of interpretative front-of-package labeling systems on beverage calories and snack calories selected, by education level[a]**

| | Beverage calories selected | | | | |
|---|---|---|---|---|---|
| | Lower education *n*=3,929 | | Higher education *n*=4,014 | | $\chi^2$, ***p*** **for interaction** |
| **Interpretative labeling system** | **ADE** | **(95% CI)** | **ADE** | **(95% CI)** | |
| Green | -33.1 | (-44.5, -21.7) | -32.4 | (-43.4, -21.4) | |
| Single traffic light | -35.0 | (-46.5, -23.6) | -25.5 | (-36.7, -14.3) | $\chi^2(8)=3.76$, $p=0.88$ |
| Physical activity | -42.4 | (-53.6, -31.2) | -34.6 | (-45.7, -23.5) | |
| Nutrient warning | -28.1 | (-39.5, -16.8) | -26.1 | (-37.3, -15.0) | |

| | Snack calories selected | | | | |
|---|---|---|---|---|---|
| | Lower education *n*=3,931 | | Higher education *n*=4,014 | | **F, *p* for interaction** |
| **Interpretative labeling system** | **ADE** | **(95% CI)** | **ADE** | **(95% CI)** | |
| Green | -13.9 | (-20.4, -7.4) | -11.4 | (-17.8, -4.9) | |
| Single traffic light | -13.9 | (-20.4, -7.4) | -13.1 | (-19.6, -6.6) | $F(4, 7935)=1.88$, $p=0.11$ |
| Physical activity | -14.3 | (-20.7, -7.9) | -22.7 | (-29.3, -16.2) | |
| Nutrient warning | -11.6 | (-18.0, -5.1) | -16.5 | (-23.0, -10.0) | |

[a]Table shows impact of each interpretative labeling system vs. control on beverage calories (top panel) and snack calories (bottom panel) selected in the trial for participants with lower education level (some college or less, left columns) and higher education level (associate degree or more, right columns). *P*-values are for the joint significance of the interaction terms between labeling arm and education level. Sample sizes vary slightly across outcomes due to missing data (*n*=2 for beverage selections [<0.1%]).

© 2023 Grummon AH et al. *JAMA Netw Open.*

## eTable 8. Effects of front-of-package labeling systems on perceptions and label reactions, n=7,945[a]

| | Green labels *n*=1,587 vs. control *n*=1,593 | | | Single traffic light labels *n*=1,588 vs. control *n*=1,593 | | | Physical activity labels *n*=1,590 vs. control *n*=1,593 | | | Nutrient warning labels *n*=1,587 vs. control *n*=1,593 | | |
|---|---|---|---|---|---|---|---|---|---|---|---|---|
| | ADE | (95% CI) | Corr. *p* | ADE | (95% CI) | Corr. *p* | ADE | (95% CI) | Corr. *p* | ADE | (95% CI) | Corr. *p* |
| **Label reactions** | | | | | | | | | | | | |
| Noticed label | **9.8%[a]** | **(6.5%, 13.2%)** | **<.001** | **11.0%[a,b]** | **(7.7%, 14.4%)** | **<.001** | **14.7%[b]** | **(11.4%, 18.1%)** | **<.001** | **26.8%[c]** | **(23.5%, 30.2%)** | **<.001** |
| Attention to label | **-0.08[a]** | **(-0.15, -0.01)** | **.02** | **0.13[b]** | **(0.06, 0.20)** | **<.001** | **0.19[b]** | **(0.12, 0.25)** | **<.001** | **0.20[b]** | **(0.13, 0.27)** | **<.001** |
| Thinking about health effects | **-0.10[a]** | **(-0.17, -0.04)** | **.003** | **0.13[b]** | **(0.06, 0.20)** | **<.001** | **0.20[b,c]** | **(0.13, 0.27)** | **<.001** | **0.27[c]** | **(0.20, 0.34)** | **<.001** |
| Negative emotions | **-0.24[a]** | **(-0.31, -0.17)** | **<.001** | **0.09[b]** | **(0.02, 0.15)** | **.01** | **0.18[c]** | **(0.11, 0.25)** | **<.001** | **0.34[d]** | **(0.28, 0.41)** | **<.001** |
| Discouragement from buying unhealthy foods | **-0.16[a]** | **(-0.23, -0.09)** | **<.001** | **0.11[b]** | **(0.04, 0.18)** | **.001** | **0.27[c]** | **(0.20, 0.34)** | **<.001** | **0.36[d]** | **(0.29, 0.43)** | **<.001** |
| Encouragement to buy healthy foods | -0.01[a] | (-0.08, 0.06) | .85 | **0.15[b]** | **(0.08, 0.22)** | **<.001** | **0.24[c]** | **(0.17, 0.31)** | **<.001** | **0.27[c]** | **(0.20, 0.34)** | **<.001** |
| Label influenced beverage choice | **-4.9%[a]** | **(-8.3%, -1.5%)** | **.009** | 2.6%[b] | (-0.8%, 6.1%) | .13 | **9.3%[c]** | **(5.9%, 12.8%)** | **<.001** | **16.4%[d]** | **(13.0%, 19.8%)** | **<.001** |
| Label influenced snack choice | -2.4%[a] | (-5.7%, 0.8%) | .14 | **6.8%[b]** | **(3.4%, 10.2%)** | **<.001** | **14.0%[c]** | **(10.6%, 17.4%)** | **<.001** | **19.6%[d]** | **(16.2%, 23.0%)** | **<.001** |
| Message reactance | **0.12[a]** | **(0.05, 0.19)** | **.001** | **0.24[b]** | **(0.17, 0.31)** | **<.001** | **0.11[a]** | **(0.04, 0.18)** | **.003** | **0.15[a]** | **(0.08, 0.22)** | **<.001** |
| **Healthfulness perceptions** | | | | | | | | | | | | |
| Healthfulness of healthy beverage | **0.23[a]** | **(0.16, 0.30)** | **<.001** | **0.28[a]** | **(0.21, 0.35)** | **<.001** | **0.29[a]** | **(0.22, 0.35)** | **<.001** | **0.20[a]** | **(0.13, 0.27)** | **<.001** |
| Healthfulness of moderately healthy beverage | **-0.13[a]** | **(-0.20, -0.06)** | **<.001** | **-0.36[b]** | **(-0.43, -0.29)** | **<.001** | **-0.12[a]** | **(-0.19, -0.05)** | **.002** | -0.03[c] | (-0.10, 0.04) | .43 |
| Healthfulness of unhealthy beverage | **-0.14[a]** | **(-0.21, -0.08)** | **<.001** | **-0.61[b]** | **(-0.67, -0.54)** | **<.001** | **-0.28[c]** | **(-0.34, -0.21)** | **<.001** | **-0.50[d]** | **(-0.57, -0.44)** | **<.001** |
| Healthfulness of healthy snack | **0.17[a]** | **(0.10, 0.24)** | **<.001** | **0.33[b]** | **(0.26, 0.40)** | **<.001** | **-0.33[c]** | **(-0.40, -0.26)** | **<.001** | **-0.14[d]** | **(-0.21, -0.07)** | **<.001** |
| Healthfulness of moderately healthy snack | -0.06[a,b] | (-0.12, 0.01) | .23 | 0.02[a] | (-0.05, 0.09) | .52 | -0.08[b] | (-0.15, -0.01) | .06 | **-0.30[c]** | **(-0.37, -0.23)** | **<.001** |
| Healthfulness of unhealthy snack | 0.00[a] | (-0.07, 0.06) | .90 | **-0.25[b]** | **(-0.32, -0.19)** | **<.001** | -0.08[a] | (-0.14, -0.01) | .06 | **-0.29[b]** | **(-0.36, -0.22)** | **<.001** |
| **Stigma** | | | | | | | | | | | | |
| Label makes me feel personally stigmatized | -0.03[a] | (-0.10, 0.04) | .36 | **0.16[b,c]** | **(0.09, 0.23)** | **<.001** | **0.10[b]** | **(0.03, 0.17)** | **.01** | **0.19[c]** | **(0.12, 0.26)** | **<.001** |
| Label stigmatizes people with obesity | **-0.21[a]** | **(-0.27, -0.14)** | **<.001** | **0.13[b,c]** | **(0.06, 0.19)** | **<.001** | 0.06[b] | (-0.01, 0.12) | .11 | **0.19[c]** | **(0.13, 0.26)** | **<.001** |
| Disgust toward people with obesity | -0.05[a] | (-0.12, 0.02) | .69 | -0.01[a] | (-0.08, 0.06) | .81 | -0.02[a] | (-0.09, 0.05) | >.99 | 0.04[a] | (-0.03, 0.11) | .70 |
| **Positive label perceptions** | | | | | | | | | | | | |
| Learned something new from the label | **0.09[a]** | **(0.02, 0.16)** | **.009** | **0.31[b]** | **(0.25, 0.38)** | **<.001** | **0.62[c]** | **(0.55, 0.69)** | **<.001** | **0.41[d]** | **(0.35, 0.48)** | **<.001** |

© 2023 Grummon AH et al. *JAMA Netw Open.*

| | | | | | | | | | | | | |
|---|---|---|---|---|---|---|---|---|---|---|---|---|
| Trust information in the label | **-0.24[a]** | **(-0.30, -0.17)** | **<.001** | **-0.18[a]** | **(-0.25, -0.11)** | **<.001** | **-0.08[b]** | **(-0.15, -0.01)** | **.03** | **0.09[c]** | **(0.02, 0.16)** | **.02** |
| Label increased perceptions of control over healthy eating decisions (yes/no) | **-5.0%[a]** | **(-8.5%, -1.6%)** | **.009** | 2.0%[b] | (-1.5%, 5.4%) | .27 | **7.9%[c]** | **(4.4%, 11.3%)** | **<.001** | **11.1%[c]** | **(7.7%, 14.6%)** | **<.001** |
| Used label to help choose beverage | **-0.13[a]** | **(-0.20, -0.06)** | **<.001** | 0.03[b] | (-0.04, 0.10) | .41 | **0.16[c]** | **(0.09, 0.22)** | **<.001** | **0.33[d]** | **(0.26, 0.40)** | **<.001** |
| Used label to help choose snack | -0.04[a] | (-0.11, 0.03) | .24 | **0.12[b]** | **(0.05, 0.19)** | **<.001** | **0.25[c]** | **(0.18, 0.32)** | **<.001** | **0.41[d]** | **(0.34, 0.48)** | **<.001** |

[a]ADE, average differential effect; Corr. *p*, corrected p-value. Continuous dependent variables were standardized prior to analyses. **Bold** indicates a statistically significant impact of the interpretative front-of-package labeling arm (green, single traffic light, physical activity, or nutrient warning label) compared to the control arm, Holm-Bonferroni-corrected $p<.05$ (4 tests per outcome, each interpretative label vs. control). ADEs sharing a superscript did not differ from one another, Holm-Bonferroni-corrected $p \geq 0.05$ (6 tests to compare the effects of the 4 interpretive labels to one another).

© 2023 Grummon AH et al. *JAMA Netw Open.*

## eTable 9. Effects of interpretative front-of-package labeling systems on stigma outcomes, by obesity status[a]

| | Perceived personal stigma | | | | |
|---|---|---|---|---|---|
| | BMI<30 kg/m$^2$ *n*=5,416 | | BMI≥30 kg/m$^2$ *n*=2,514 | | F, *p* for interaction |
| **Interpretative labeling system** | **ADE** | **(95% CI)** | **ADE** | **(95% CI)** | |
| Green | 0.02 | (-0.08, 0.12) | -0.16 | (-0.30, -0.01) | F(4, 7920)=1.42, *p*=0.22 |
| Single traffic light | 0.24 | (0.14, 0.34) | 0.10 | (-0.05, 0.24) | |
| Physical activity | 0.14 | (0.04, 0.23) | 0.08 | (-0.06, 0.23) | |
| Nutrient warning | 0.28 | (0.18, 0.38) | 0.13 | (-0.01, 0.27) | |

| | Perceived obesity stigma | | | | |
|---|---|---|---|---|---|
| | BMI<30 kg/m$^2$ *n*=5,417 | | BMI≥30 kg/m$^2$ *n*=2,514 | | F, *p* for interaction |
| **Interpretative labeling system** | **ADE** | **(95% CI)** | **ADE** | **(95% CI)** | |
| Green | -0.24 | (-0.34, -0.14) | -0.24 | (-0.39, -0.10) | F(4, 7921)=0.17, *p*=0.95 |
| Single traffic light | 0.17 | (0.07, 0.27) | 0.12 | (-0.03, 0.26) | |
| Physical activity | 0.08 | (-0.02, 0.18) | 0.04 | (-0.10, 0.19) | |
| Nutrient warning | 0.26 | (0.15, 0.36) | 0.21 | (0.06, 0.35) | |

| | Disgust toward people with obesity | | | | |
|---|---|---|---|---|---|
| | BMI<30 kg/m$^2$ *n*=5,416 | | BMI≥30 kg/m$^2$ *n*=2,514 | | F, *p* for interaction |
| **Interpretative labeling system** | **ADE** | **(95% CI)** | **ADE** | **(95% CI)** | |
| Green | -0.01 | (-0.11, 0.09) | -0.15 | (-0.29, -0.01) | F(4, 7920)=1.60, *p*=0.17 |
| Single traffic light | 0.00 | (-0.10, 0.10) | -0.03 | (-0.18, 0.11) | |
| Physical activity | 0.01 | (-0.09, 0.11) | -0.11 | (-0.25, 0.04) | |
| Nutrient warning | 0.12 | (0.02, 0.22) | -0.07 | (-0.22, 0.07) | |

[a]*Abbreviations*. BMI, body mass index. Table shows impact of each interpretative labeling system vs. control on perceived personal stigma (top panel), perceived obesity stigma (middle panel) and disgust toward people with obesity (bottom panel) for participants with BMI>30 kg/m$^2$ (left columns) and ≥30 kg/m$^2$ (right columns). *P*-values are for the joint significance of the interaction terms between labeling arm and obesity status. Sample sizes vary slightly across outcomes due to missing data (*n*=1 for perceived personal stigma [<0.1%] and *n*=1 for disgust toward people with obesity [<0.1%]).

© 2023 Grummon AH et al. *JAMA Netw Open.*

## eReferences


Acton, R., & Hammond, D. (2018). Do consumers think front-of-package "high in" warnings are harsh or reduce their control? A test of food industry concerns. *Obesity (Silver Spring)*, *26*(11), 1687–1691. https://doi.org/10.1002/oby.22311

Adjoian, T. K., Firestone, M. J., Eisenhower, D., & Yi, S. S. (2016). Validation of self-rated overall diet quality by Healthy Eating Index-2010 score among New York City adults, 2013. *Preventive Medicine Reports*, *3*, 127–131. https://doi.org/10.1016/j.pmedr.2016.01.001

Baig, S. A., Noar, S. M., Gottfredson, N. C., Boynton, M. H., Ribisl, K. M., & Brewer, N. T. (2019). UNC Perceived Message Effectiveness: Validation of a brief scale. *Annals of Behavioral Medicine*, *53*(8), 732–742. https://doi.org/10.1093/abm/kay080

Bollard, T., Maubach, N., Walker, N., & Mhurchu, C. N. (2016). Effects of plain packaging, warning labels, and taxes on young people's predicted sugar-sweetened beverage preferences: An experimental study. *International Journal of Behavioral Nutrition and Physical Activity*, *13*(1), 95.

Brewer, N., Parada Jr., H., Hall, M., Boynton, M., Noar, S., & Ribisl, K. (2019). Understanding why pictorial cigarette pack warnings increase quit attempts. *Annals of Behavioral Medicine*, *53*(3), 232–243. https://doi.org/10.1093/abm/kay032

Centers for Disease Control and Prevention National Center for Health Statistics. (2016). *National Health and Nutrition Examination Survey 1999-2016 Survey Content Brochue*. National Center for Health Statistics. https://www.cdc.gov/nchs/data/nhanes/survey_content_99_16.pdf

Corvalán, C., Reyes, M., Garmendia, M. L., & Uauy, R. (2013). Structural responses to the obesity and non-communicable diseases epidemic: The C hilean L aw of F ood L abeling and A dvertising. *Obesity Reviews*, *14*, 79–87.

Fathelrahman, A. I., Omar, M., Awang, R., Cummings, K. M., Borland, R., & Samin, A. S. B. M. (2010). Impact of the new Malaysian cigarette pack warnings on smokers' awareness of health risks and interest in quitting smoking. *International Journal of Environmental Research and Public Health*, *7*(11), 4089–4099. https://doi.org/10.3390/ijerph7114089

Gibson, L., Stephens-Shields, A., Hua, S., Orr, J., Lawman, H., Bleich, S., Volpp, K., Bleakley, A., Thorndike, A., & Roberto, C. (2022, November). *A Randomized Field Experiment Comparing Nutrition and Tax Salience*

*Messages on Vending Machine Sales* [Oral Presentation]. ObesityWeek, San Diego, CA. https://onlinelibrary.wiley.com/doi/full/10.1002/oby.23625

Grummon, A. H., & Hall, M. G. (2020). Sugary drink warnings: A meta-analysis of experimental studies. *PLOS Medicine*, *17*(5), e1003120. https://doi.org/10.1371/journal.pmed.1003120

Hall, M. G., Sheeran, P., Noar, S. M., Ribisl, K. M., Boynton, M. H., & Brewer, N. T. (2017). A brief measure of reactance to health warnings. *Journal of Behavioral Medicine*, *40*(3), 520–529. https://doi.org/10.1007/s10865-016-9821-z

Hammond, D., Fong, G. T., McDonald, P. W., Cameron, R., & Brown, K. S. (2003). Impact of the graphic Canadian warning labels on adult smoking behaviour. *Tobacco Control*, *12*(4), 391–395.

Hayward, L., & Vartanian, L. (2019). Potential unintended consequences of graphic warning labels on sugary drinks: Do they promote obesity stigma? *Obesity Science & Practice*, *5*(4), 333–341.

Nonnemaker, J., Farrelly, M., Kamyab, K., Busey, A., & Mann, N. (2010). *Experimental study of graphic cigarette warning labels* (Final Results Report RTI Project Number 0212305.007.003). RTI International. https://www.fda.gov/media/136126/download

Puhl, R., Luedicke, J., & Lee Peterson, J. (2013). Public Reactions to Obesity-Related Health Campaigns: A Randomized Controlled Trial. *American Journal of Preventive Medicine*, *45*(1), 36–48. https://doi.org/10.1016/j.amepre.2013.02.010

Roberto, C. A., Wong, D., Musicus, A., & Hammond, D. (2016). The influence of sugar-sweetened beverage health warning labels on parents' choices. *Pediatrics*, *137*(2), e20153185. https://doi.org/10.1542/peds.2015-3185

RTI International. (2018). *Experimental Study on Warning Statements for Cigarette Graphic Health Warnings: Study 1 Report* (FDA Report February 2020). https://www.fda.gov/media/136127/download

Semega, J., & Kollar, M. (2022). *Income in the United States: 2021* (No. P60-276; Current Population Reports). United States Census Bureau. https://www.census.gov/library/publications/2022/demo/p60-276.html

Thomson, J., Landry, A., & Walls, T. (2022). Can United States Adults Accurately Assess Their Diet Quality? *Current Developments in Nutrition*, *6*(Suppl 1), 952. https://doi.org/10.1093/cdn/nzac067.072

United States Census Bureau. (2022, September 22). *Accessing PUMS Data*. American Community Survey (ACS). https://www2.census.gov/programs-surveys/acs/data/pums/2021/1-Year/

© 2023 Grummon AH et al. *JAMA Netw Open.*
