# Supplementary material for: Effects of 4 Interpretive Front-of-Package Labeling Systems on Hypothetical Beverage and Snack Selections: A Randomized Clinical Trial
Source: JAMA Netw Open. 2023 Sep 13;6(9):e2333515. doi: 10.1001/jamanetworkopen.2023.33515 (PMC10500374; doi:10.1001/jamanetworkopen.2023.33515)
Supplement: Supplement 3. — Data Sharing Statement [file jamanetwopen-e2333515-s003.pdf]

Data Sharing Statement

Grummon. Effects of 4 Interpretive Front-of-Package Labeling Systems on Hypothetical Beverage and Snack Selections. *JAMA Netw Open*. Published September 13, 2023. doi:10.1001/jamanetworkopen.2023.33515

Data
Data available: Yes
Data types: Deidentified participant data
How to access data: We will upload all data to a public ResearchBox repository upon publication (https://researchbox.org/1895).
When available: With publication

Supporting Documents
Document types: None

Additional Information
Who can access the data: Anyone requesting the data will be able to download them.
Types of analyses: For any purpose
Mechanisms of data availability: Data will be publicly available to download.
Any additional restrictions: NA
